# Mucosal-Associated Invariant T (MAIT) cells are highly activated in duodenal tissue of humans with *Vibrio cholerae* O1 infection: A preliminary report

**DOI:** 10.1371/journal.pntd.0010411

**Published:** 2022-05-12

**Authors:** Taufiqur R. Bhuiyan, M. Arifur Rahman, Shubhanshi Trivedi, Taliman Afroz, Hasan Al Banna, Mohammad Rubel Hoq, Ioana Pop, Owen Jensen, Rasheduzzaman Rashu, Muhammad Ikhtear Uddin, Motaher Hossain, Ashraful I. Khan, Fahima Chowdhury, Jason B. Harris, Stephen B. Calderwood, Edward T. Ryan, Firdausi Qadri, Daniel T. Leung

**Affiliations:** 1 International Centre for Diarrhoeal Disease Research, Bangladesh, Dhaka, Bangladesh; 2 Division of Infectious Diseases, Department of Internal Medicine, University of Utah School of Medicine, Salt Lake City, Utah, United States of America; 3 Division of Microbiology and Immunology, Department of Pathology, University of Utah School of Medicine, Salt Lake City, Utah, United States of America; 4 Division of Infectious Diseases, Massachusetts General Hospital, Boston, Massachusetts, United States of America; 5 Department of Pediatrics, MassGeneral Hospital for Children, Boston, Massachusetts, United States of America; 6 Division of Pediatric Global Health, Massachusetts General Hospital, Boston, Massachusetts, United States of America; 7 Department of Medicine, Harvard Medical School, Boston, Massachusetts, United States of America; 8 Department of Immunology and Infectious Diseases, Harvard T.H. Chan School of Public Health, Boston, Massachusetts, United States of America; Tel Aviv University Sackler Faculty of Medicine, ISRAEL

## Abstract

Mucosal-associated invariant T (MAIT) cells are unconventional T lymphocytes with a semi-conserved TCRα, activated by the presentation of vitamin B metabolites by the MHC-I related protein, MR1, and with diverse innate and adaptive effector functions. The role of MAIT cells in acute intestinal infections, especially at the mucosal level, is not well known. Here, we analyzed the presence and phenotype of MAIT cells in duodenal biopsies and paired peripheral blood samples, in patients during and after culture-confirmed *Vibrio cholerae* O1 infection. Immunohistochemical staining of duodenal biopsies from cholera patients (n = 5, median age 32 years, range 26–44, 1 female) identified MAIT cells in the lamina propria of the crypts, but not the villi. By flow cytometry (n = 10, median age 31 years, range 23–36, 1 female), we showed that duodenal MAIT cells are more activated than peripheral MAIT cells (p < 0.01 across time points), although there were no significant differences between duodenal MAIT cells at day 2 and day 30. We found fecal markers of intestinal permeability and inflammation to be correlated with the loss of duodenal (but not peripheral) MAIT cells, and single-cell sequencing revealed differing T cell receptor usage between the duodenal and peripheral blood MAIT cells. In this preliminary report limited by a small sample size, we show that MAIT cells are present in the lamina propria of the duodenum during *V*. *cholerae* infection, and more activated than those in the blood. Future work into the trafficking and tissue-resident function of MAIT cells is warranted.

## Author summary

Mucosal-associated invariant T (MAIT) cells are unconventional T cells with characteristics of both the innate and adaptive arms of the immune system. The role of MAIT cells during gut infections is not well known. In this study, we looked at MAIT cells in cholera patients, both in the blood and in tissue biopsies from a special section of the gut called the “duodenum”. We used both microscope-(n = 5, median age 32 years, 1 female) and flow cytometry-(n = 10, median age 31 years, 1 female) based methods to demonstrate the presence of MAIT cells in the gut. We found primarily that MAIT cells in the gut are more activated that those in the blood, and found an association between loss of gut MAIT cells and stool markers of gut inflammation. We also showed that the MAIT cells in the gut have a different fingerprint than those in the blood. In this preliminary report limited by a small sample size, we show the presence of MAIT cells in the gut, and that they are more activated than the ones in the blood during cholera infection.

## Introduction

Mucosal-associated invariant T (MAIT) cells are a recently identified non-conventional T cell subset. They express an invariant T cell receptor (TCR) Vα chain (Vα7.2 or TRAV1-2 in humans) and a variable but restricted number of TCRβ chains. MAIT cells are found in mucosal tissues and associated organs, including the liver, lung, mesenteric lymph nodes, and intestinal epithelium [1]. In human peripheral blood, MAIT cells constitute approximately 1–10% of total T lymphocytes [2], and they account for up to 40% of T cells in the liver [3]. In the human intestine, they are located in both the lamina propria and as part of the intraepithelial lymphocyte compartment [4]. The antigen for MAIT cells has been identified as belonging to a class of transitory intermediates of the riboflavin synthesis pathway [5], which are produced by many, but not all, bacteria and yeast. These vitamin B metabolites are presented on the surface of MR1 [6], the non-polymorphic MHC class I related protein. MAIT cells are capable of releasing IFN-γ, TNF, and IL-17 in response to stimulation, and they also possess cytotoxic activity [7, 8], killing infected cells via granzyme B and perforin.

Cholera is a life-threatening diarrheal disease caused primarily by *Vibrio cholerae* O1, responsible for close to 3 million cases and 100,000 deaths annually in endemic countries alone [9]. The mechanisms of protection against cholera are not well understood, although we have previously shown that in patients hospitalized with severe cholera, both adaptive and innate immune responses are induced. We have demonstrated increases in circulating *V*. *cholerae* antigen-specific antibodies, as well as antigen-specific memory B and memory T cell responses in both children and adults after cholera infection [10, 11]. We have also shown, through endoscopically-obtained duodenal biopsies, that there is an increase in cells of the innate immune system and their mediators during acute cholera [12, 13].

We have previously reported that circulating MAIT cells are activated during *V*. *cholerae* O1 infection, and that in children, but not adults, their circulating numbers are significantly decreased by day 7 after infection and onward [14]. We also demonstrated an association between circulating MAIT cells and class-switched antibody responses against V. cholerae lipopolysaccharide (LPS). Despite their abundance in the intestinal mucosa, little is known regarding the activity of MAIT cells in mucosal tissue during acute enteric infection. Thus, our objective was to characterize MAIT cells in the intestinal mucosa during *V*. *cholerae* infection.

## Methods

### Ethics

This study was approved by the Ethical Review and Research Review Committees of the International Centre for Diarrhoeal Disease Research, Bangladesh (icddr,b), and the Institutional Review Boards of Massachusetts General Hospital and the University of Utah.

### Study population and sample collection

We enrolled patients admitted to the Dhaka Hospital of the icddr,b who had acute watery diarrhea and positive stool cultures for *V*. *cholerae* O1. We recruited patients who had no underlying medical conditions and had an otherwise normal physical examination and baseline laboratory parameters. All patients were rehydrated and provided antibiotics per hospital protocol (a minimum of 18–24 hours prior to enrollment) and were hemodynamically stable at the time of the procedure. After written informed consent, we performed esophagogastroduodenoscopy (EGD) on day 2 (acute illness phase), day 30 (convalescent phase), and day 180 (late convalescent phase) following admission. Using standard forceps, we obtained approximately six duodenal pinch biopsies (from second part of duodenum) of approximately 1 mm^3^ in diameter from each patient at each time point. We also obtained a stool sample from each patient at day 2 and venous blood samples at days 2, 7, 30 and 180. To characterize MAIT cell presence and activity in the duodenum during cholera, we obtained duodenal biopsies from a total of 15 cholera patients, on 10 of whom we performed flow cytometric (FC) analysis and 5 of whom we performed immunohistochemical (IHC) analysis (S1 Table). The median age of the FC group was 31 (range 23 to 36) years, and the median age of the IHC group was 30 (range 26–44) years. Only one female was recruited for each group. All patients mounted at least a 16-fold rise in vibriocidal response by day 7 of illness. These patients were from two larger cohorts named “SEGD” and “PIC”, both of which had paired biopsy and blood specimens. However, histology was performed from “PIC” patients only and flow cytometry was done in “SEGD” patients only (S1 Table).

### Phenotyping of MAIT cells by flow cytometry

From venous blood samples, we isolated peripheral blood mononuclear cells (PBMCs) by differential centrifugation on Ficoll-Isopaque (Pharmacia, Piscataway, NJ). We stored plasma at -80°C for use in immunological assays as detailed below. From duodenal biopsy samples, we isolated lamina propria lymphocytes (LPLs) by incubation in 1 mM EDTA and 1 mM dithiothreitol (DTT) followed by filtering through a nylon cell strainer and treatment with collagenase and DNase, as we have previously described [15]. We washed and stained the freshly isolated PBMCs and LPLs with an established fluorochrome-conjugated antibody panel designed for MAIT cell isolation. Antibodies (S2 Table) were purchased from BioLegend (San Diego, CA), BD Biosciences (San Jose, CA), or Life Technologies (Carlsbad, CA): TCR Vα7.2-PE (Clone: 3C10, Cat# 351706, Biolegend), CD3-PE-Texas Red (Clone: 7D6, Cat# MHCD0317, Life technologies), CD4-Amcyan (Clone: SK3, Cat# 339187, BD), CD8-FITC (Clone: RPA-T8, Cat# 561948, BD Pharmingen), CD161-APC (Clone: DX12, Cat# 550968, BD Pharmingen), CD38-PE-Cy7 (Clone: HIT2, Cat# 303516, Biolegend), CD69-PerCP-Cy5.5 (Clone: FN50, Cat# 560738, BD Pharmingen), and DAPI (Cat# 564907, BD Pharmingen). After 45 minutes incubation at 4°C, we analyzed at least 10^5^ lymphocytes on a FACSAria III flow cytometer (BD Biosciences, San Jose, CA) and analyzed data using FlowJo 10 software (TreeStar Inc, Ashland, OR). We used Cytometer Setup & Tracking beads (BD Biosciences) to check for inter-day variability, and Fluorescence Minus One (FMO) controls. We defined MAIT cells as live (DAPI^−^) CD3^+^CD161^hi^Vα7.2^+^ cells, expressed as a percentage of total CD3+ lymphocytes, and used CD38 and CD69 as markers of cell activation.

### Immunohistochemistry

In a separate set of patients, we embedded cryosections from duodenal biopsies in Tissue Tek OCT compound (Sakura USA, Torrance, CA) and used a Leica CM3000 Cryostat (Leica Instruments GmbH, Nussloch, Germany) to cut 5 μm sections, picked up on poly-L-lysine coated slides, and air-dried. In line with immunohistochemical methods from reports available at time of the experiments [16, 17], we stained the sections with primary antibodies against CD3, IL-18Rα, and Vα7.2, followed by corresponding secondary antibodies conjugated to: AF555, AF488, and Cy5. Antibodies were obtained from Dako (Carpinteria, CA), BioLegend, or LifeTechnologies. We counterstained with DAPI to visualize cell nuclei. We used the Nuance Multispectral Imaging system (CRI, Woburn, MA) to visualize and captured images with a digital camera. We analyzed the images with ImageJ software (US National Institutes of Health, Bethesda, MD). We defined MAIT cells as CD3^+^IL-18Rα^+^Vα7.2^+^ cells, as described previously [16]. For each patient, a single unblinded operator, using ImageJ software, enumerated both MAIT and CD3+ cells from a single biopsy section at each time point from paired (available at both day 2 and day 30) samples.

### Vibriocidal and plasma antibody levels

To examine the vibriocidal antibody response to the two serotypes of *V*. cholerae O1 (Ogawa and Inaba), we performed the vibriocidal assay as previously described [18]. We quantified LPS (prepared from *V*. *cholerae* O1 as previously described [18])-specific IgA, IgG, and IgM antibody responses in plasma using a kinetic ELISA method [19].

### Markers of intestinal inflammation and permeability

In a subset of patients from the flow cytometry cohort whom we had data from both day 2 and day 30, we performed ELISA to determine the concentration of myeloperoxidase (MPO; Alpco, Salem, NH) and alpha anti-trypsin (AAT; ImmuChrom GmBH, NC) from stool samples obtained at the time of admission (day 0), at dilution factors of 1:200 and 1:400, respectively.

### Single Cell TCR sequencing

MAIT cells from PBMCs and LPLs from one donor, at acute stages of infection (days 2 and 7) and from a second donor at a convalescent stage of infection (day 180), were single cell sorted using the Aria II cell sorter (BD Biosciences) directly into One Step RT-PCR reaction mix (NEB) loaded in MicroAmp Optical 96-well reaction plates (Applied Biosystems). MAIT cells were defined as live (DAPI^−^) CD3^+^CD4^−^CD161^hi^Vα7.2^+^ cells. Following reverse transcription and preamplification reaction, a series of three nested PCR’s were run using primers for TCR sequence and gene expression as described earlier [20]. To separate reads from every well in every plate according to specified barcodes we processed and demultiplexed raw sequencing data using a custom software pipeline described in [20]. The data were analyzed using the R package as described [20].

### Statistics

**Fig 1:**
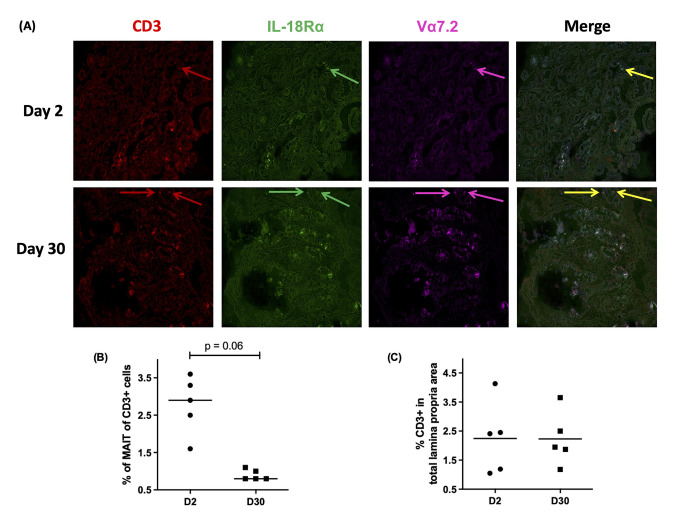
MAIT cell localization and frequency in LP by Immunohistochemistry. Cryosections of duodenal biopsies obtained from five cholera patients (n = 5) were stained with antibodies as mentioned in methods and imaged using Leica CM3000 Cryostat. (A) Representative images of duodenal biopsies at day 2 and day 30, shows CD3^+^ T cells (red arrows), IL-18Rα^+^ T cells (green arrows), Vα 7.2^+^ cells (magenta arrows), and the merged image shows MAIT cells (yellow arrows indicating CD3^+^ IL-18Rα^+^ Vα7.2^+^ cells). (B) Frequency of MAIT cells, as a % of total CD3+ cells in the duodenum at day 2 and day 30 post infection (p.i.), and (C) Frequency of CD3^+^ cells, as % of total cells in the lamina propria area at day 2 and day 30 p.i. Statistical significance of the difference between day 2 and day 30 was determined using the Wilcoxon signed-ranked test.

**Fig 2:**
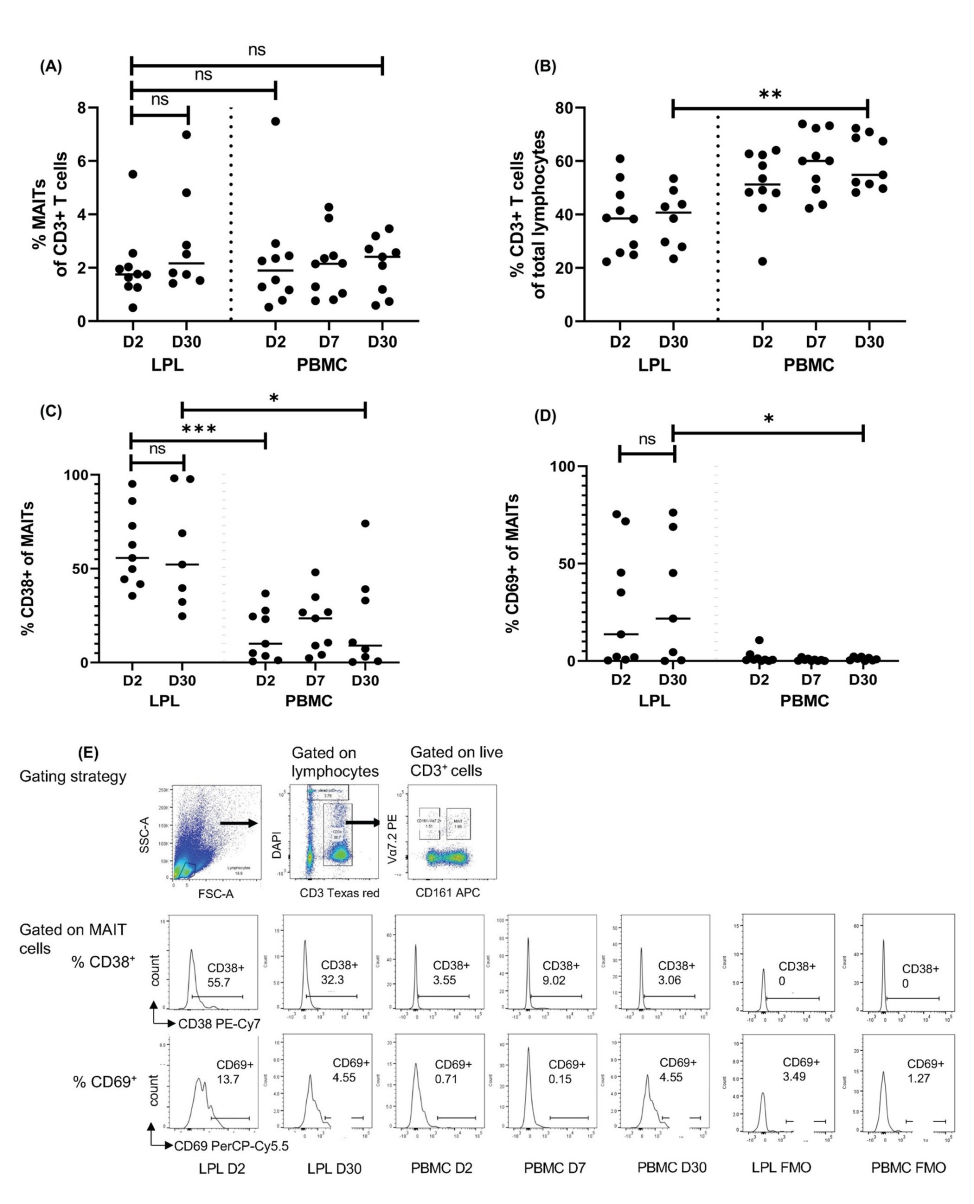
MAIT cell frequency and activation in PBMCs and LPLs by flow cytometric analysis. Lamina propria lymphocytes (LPLs) and PBMCs were isolated from patients. (A) Frequency of MAIT cells (live (DAPI^−^) CD3^+^CD161^hi^Vα7.2^+^ cells), as % of total CD3^+^ lymphocytes, and (B) Frequency of CD3^+^ T cells, as of total lymphocyte population, in LPLs at day 2 (n = 10) and day 30 (n = 8) p.i., and PBMCs, at day 2 (n = 10), day 7 (n = 10), and day 30 (n = 9) p.i. (C) Frequency of CD38^+^ cells, and (D) frequency of CD69^+^ cells, gated on MAIT cells in LPLs at day 2 (n = 9) and day 30 (n = 7) p.i., and PBMCs, at day 2 (n = 9), day 7 (n = 9), and day 30 (n = 8) p.i. Horizontal bar shows median values in graphs. (E) Gating strategy used to identify MAIT cells and representative histograms showing % CD38+ and % CD69+ cells gated on MAIT cells in LPL and PBMCs. Statistical significance of difference between groups was determined using one-way ANOVA with Tukey’s post hoc testing. * denotes p ≤ 0.05, ** denotes p ≤ 0.01, and *** denotes p < 0.001.

**Fig 3:**
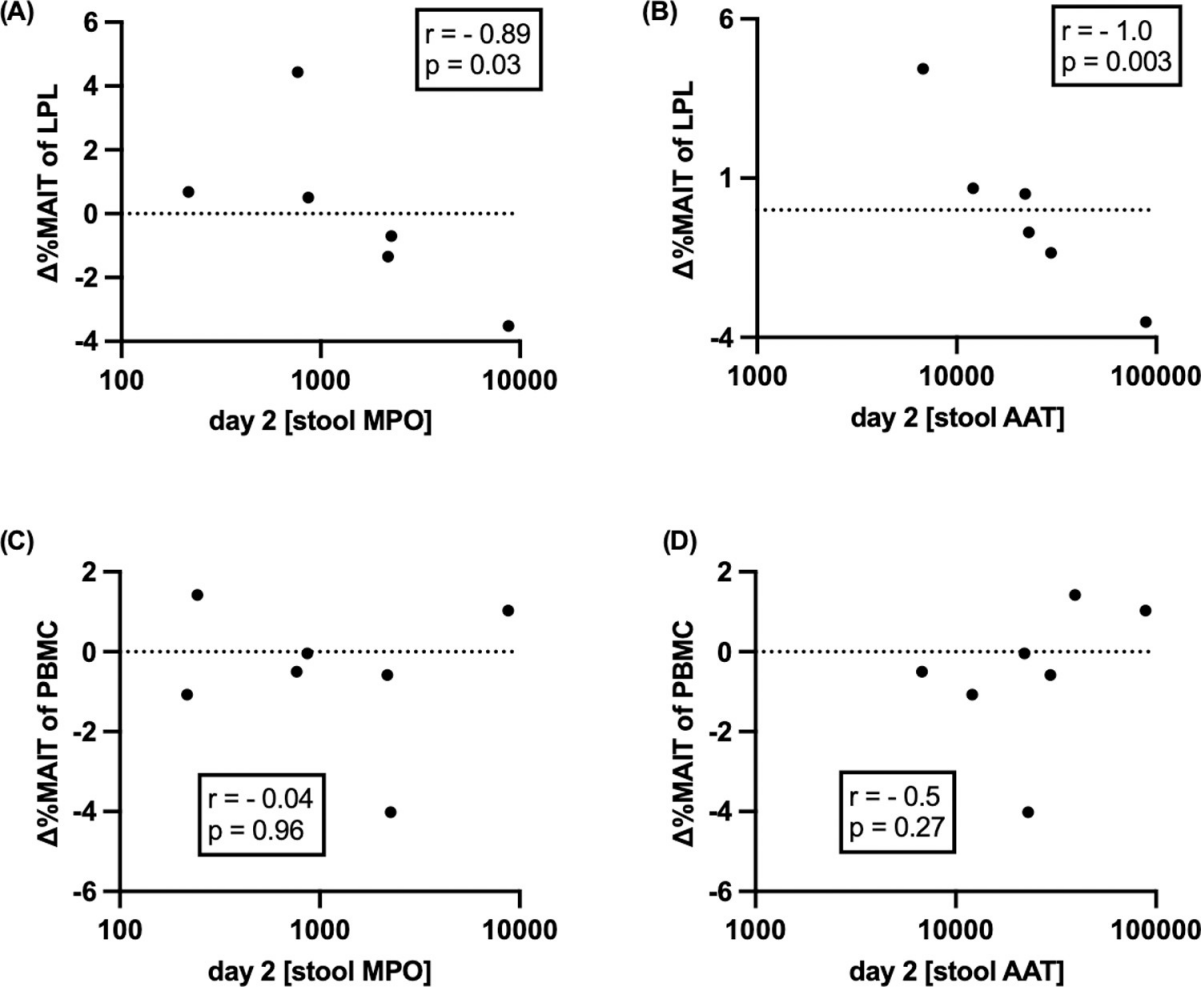
MAIT cell number and correlation with fecal markers of intestinal permeability (myeloperoxidase (MPO)) and inflammation (alpha-1-1antitrypsin (AAT)). Stool markers of intestinal permeability and inflammation were measured using ELISA, values were log transformed and the correlation with LPL MAIT cell number was determined using Spearman’s correlation. (A-B) shows the correlation of LPL MAITs with MPO (A) and with AAT (B), (n = 6). (C-D) shows the correlation of PBMC MAITs with MPO (C) and with AAT (D), (n = 7). [Δ%MAIT = day30—day2%MAIT].

We used the Wilcoxon signed-ranked test for comparisons of frequency and activation of MAIT cells between different study days (Fig 1). We used one-way ANOVA with Tukey’s multiple comparison test for comparison of two or more groups (Fig 2). We log transformed MPO and AAT values and used Spearman’s correlation to determine their association with changes in LPL MAIT cells (Fig 3). All P values were two-tailed, with a value of <0.05 considered the threshold for statistical significance. We performed analyses using STATA version 13.1 (StataCorp, College Station, TX), and GraphPad Prism version 6.0 (GraphPad Software, Inc., La Jolla, CA).

## Results

### MAIT cells are found predominantly in the lamina propria of the duodenum, and are more abundant during acute infection than at convalescence

We performed immunohistochemistry from frozen sections of paired duodenal biopsies from five cholera patients. We identified MAIT cells as CD3^+^IL-18Rα^+^Vα7.2^+^ cells (Fig 1A). The majority of MAIT cells were identified in the lamina propria of the crypt, with no cells identified in the villi. By this technique, we found that the frequency of MAIT cells, as a % of total CD3^+^ cells, was statistically non-significantly higher at day 2 of infection compared to day 30 (*p* = 0.06, Fig 1B), though this was the smallest achievable p-value with only 5 paired observations. In contrast, we demonstrated that the occurrence of CD3^+^ cells, as % of total cells in the lamina propria, did not change between acute and convalescent stages of infection (Fig 1C).

### Compared to peripheral blood MAIT cells, duodenal MAIT cells at both acute and convalescent stages are more activated, but present in similar frequencies

We performed flow cytometric analysis on LPLs isolated from duodenal biopsies in 10 patients, of which only 8 completed 30 days of follow-up. We found that during all phases of cholera, MAIT cells were present in the duodenal lamina propria at frequencies similar to those found in the periphery (Fig 2A). We demonstrated that the occurrence of CD3^+^ cells, as % of total cells in the lamina propria at the convalescent stage, is lower than in the periphery (Fig 2B). Using CD38^+^ as a marker of activation, we found that at both days 2 and 30, duodenal MAIT cells were significantly more activated than peripheral MAIT cells. At day 2, a mean of 60% of all duodenal MAITs were CD38^+^, compared to 15% of all peripheral MAIT cells (95% CI of difference 17–75, P = 0.0005); similarly, at day 30, a mean of 59% of duodenal MAIT cells were activated, compared to 21% of peripheral MAIT cells (95% CI of difference 6–70, P < 0.01) (Fig 2C). We also found that at day 30, percentage frequencies of CD69^+^ duodenal MAIT cells were significantly higher than peripheral MAIT cells (P < 0.05) (Fig 2D). We found no significant differences in CD38^+^ MAIT cells and CD69^+^ MAIT cells between duodenal MAIT cells at day 2 and day 30. Fig 2E shows the gating strategy for MAIT cells and representative flow cytometry plots of CD38^+^ and CD69^+^ MAIT cells in the lamina propria and the periphery.

### Increased intestinal permeability and inflammation are associated with loss of duodenal (but not peripheral) MAIT cells

Given the known cytotoxic capacity of MAIT cells and their activation in inflammatory bowel disease [16, 21], we examined whether baseline intestinal inflammation was associated with changes in MAIT cell frequency observed in flow cytometry. We measured two common fecal markers of intestinal permeability (myeloperoxidase (MPO) and inflammation (alpha-1-1antitrypsin (AAT)) in six of the eight cholera patients from whom we had paired days 2 and 30 LPL MAIT cell data from the flow cytometry cohort, and seven of the eight from whom we had paired PBMC data. We found a high level of correlation between the markers and the loss of duodenal MAIT cells from day 2 to 30 (r = 0.90, p = 0.03 for MPO; r = 1.00, P = 0.003 for AAT; Fig 3A and 3B). We did not find any correlation between these markers and changes in the frequency of peripheral MAIT cells (r = -0.04, p = 0.96 for MPO; r = -0.50, P = 0.27 for AAT; Fig 3C and 3D).

### Single-cell TCR analysis of duodenal MAIT cells reveals a different TCR usage than that of MAIT cells in peripheral blood during acute infection

**Fig 4:**
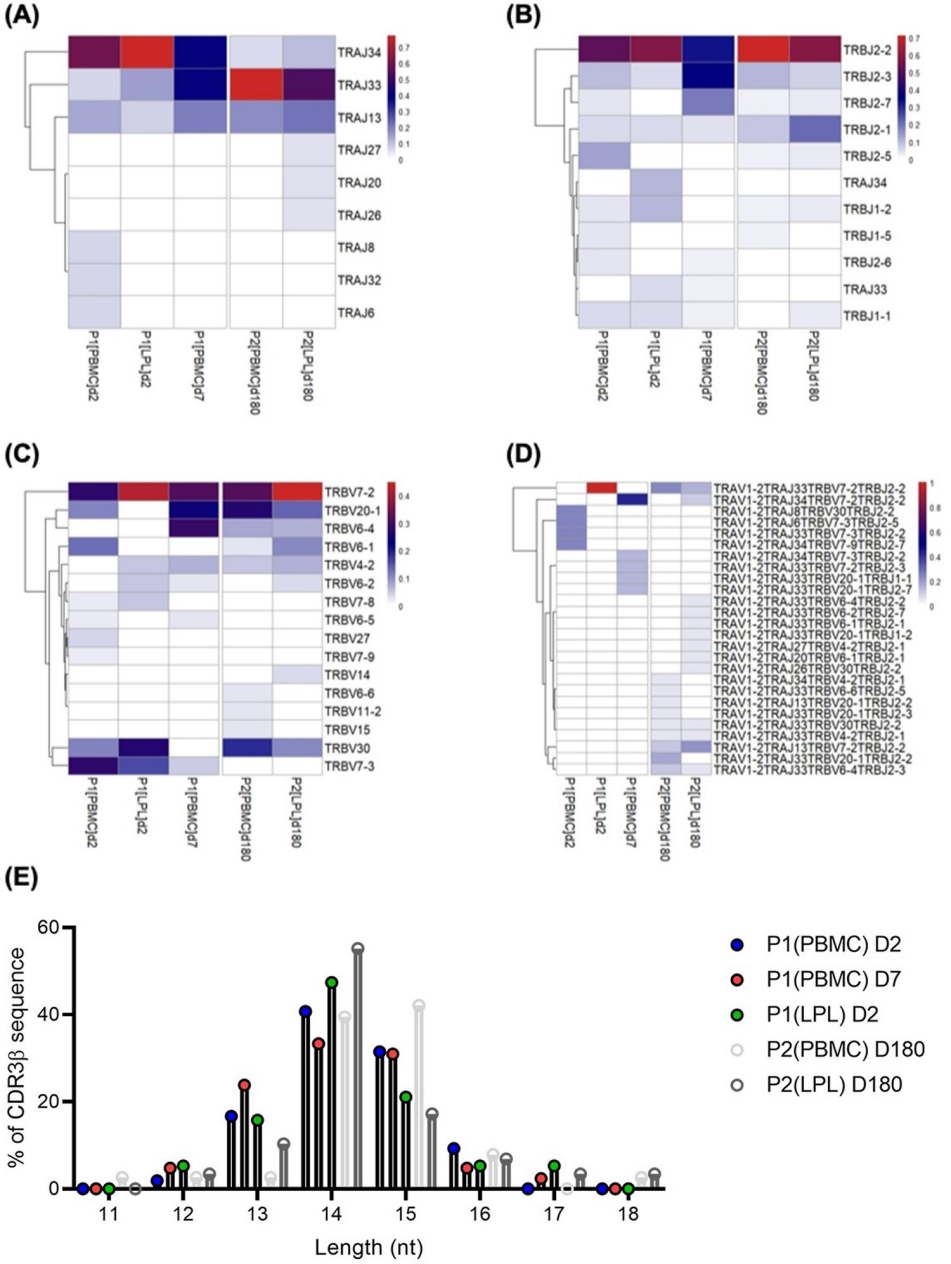
Different distribution of MAIT TCR in LPL compared to PBMCs. MAIT cells were sorted from LPL and PBMCs from two (n = 2) patients at the acute and convalescent stage of illness and TCR usage was analyzed at the single-cell level using Illumina MiSeq sequencing. (A) Paired MAIT TCRαβ usage in each patient is shown as a heat map with hierarchical clustering performed using Euclidean distance. (B, C, and D) TRAJ, TRBV and TRBJ usage in LPL and PBMCs is shown as heat map. (E) The length distribution of MAIT CDR3β sequence in LPL and PBMCs is shown as a bar graph. The x axis shows length distribution of amino acids and the y axis shows percentage frequency of CDR3β sequence found with that amino acid length.

Few studies have reported TCR usage of MAIT cells in tissues [22–24], with minimal data on paired αβ TCR usage. To understand how MAIT TCR repertoire is affected in LPLs and PBMCs during cholera, we utilized paired TCR-phenotype single-cell Illumina sequencing as previously described [20], on paired tissue-blood samples from two patients. The αβ TCR usage are shown as heatmaps in (Fig 4A–4D). When we compared TCR usage from PBMC (46 cells were sequenced at day 2 and 7) and LPL (46 cells were sequenced at day 2) samples that were available from the single patient examined during acute infection, we found differences in usage of TRAJ, TRBV, and TRBJ between the duodenal LPLs and circulating PBMCs (Fig 4A–4C). We noted that MAIT clones observed in PBMCs obtained at day 7 differed from those observed at day 2. When analyzing based on TCR β usage alone, in concurrence with previous studies [22–25], we also found that MAIT cells in PBMCs obtained at day 2 and day 7 expressed TRBV20 and TRBV6 (Fig 4C). Unfortunately, despite many unpaired TCRα and TCRβ sequences available from the acute day 2 LPL sample (Fig 4A–4C), there was only one paired TCRαβ clone sequenced (S3 Table), and thus comparisons between LPL and PBMC clonality could not be made based on paired TCR reads. On the other hand, numerous LPL-paired TCR reads were successfully sequenced for the convalescent stage (day 180) donor, for whom we sequenced 89 PBMC and 91 LPL cells and found overlapping MAIT TCR usage (Fig 4D and S3 Table), with the majority of PBMC and LPL MAIT cells expressing TRAJ33 (Fig 4A), TRBV7-2, TRBV20-1 or TRBV30 (Fig 4B), and TRBJ2-2 (Fig 4C).

MAIT TCR β-chain repertoire diversity resides within the complementarity determining region (CDR) 3β loop [26–28] and to determine if MAIT TCR usage differ at the CDR3 level, we investigated the length distribution of amino acids in the CDR3β region of PBMCs and LPLs. We observed that the percentage frequency of CDR3β sequences with 14 nucleotides was highest, and relatively higher in LPLs compared to PBMCs. In contrast, the percentage of CDR3β sequences with 15 nucleotides was relatively higher in PBMCs compared to LPL samples (Fig 4E). Overall, differential patterns of MAIT TCR usage was observed in the duodenum compared with peripheral blood during acute and convalescent infection.

## Discussion

*V*. *cholerae* infection is caused by the ingestion of bacteria, followed by colonization of the small intestine where cholera toxin is elaborated, resulting in chloride ion secretion and secretory diarrhea [29]. MAIT cells are innate-like lymphocytes known to provide immediate effector functions in response to infections in human tissues [8, 30]. Although studies have described MAIT cells in the human intestinal mucosa [4, 31, 32], there is limited data available on MAIT cells in the intestinal mucosa during an acute intestinal infection. In this study, we performed endoscopy and obtained duodenal biopsies in a cohort of patients with culture-confirmed severe dehydrating *V*. *cholerae* O1 infection. We showed that during acute human cholera, MAIT cells in the duodenal mucosa are present at frequencies similar to that seen in the peripheral circulation. Using immunohistochemistry and multispectral imaging, we demonstrated that the vast majority of MAIT cells are in the lamina propria, predominantly in the crypt, and rarely in the villi. This is consistent with a previous report using MR1 tetramer staining of healthy human jejunal tissue, showing that MAIT cells reside predominantly near the base of the villi [4].

We have previously shown, in endoscopically-obtained biopsies from cholera patients, that during acute disease, there is an upregulation of innate responses, including infiltration of neutrophils, degranulation of mast cells, and expression of pro-inflammatory cytokines [13, 33]. Using immunohistochemistry and flow cytometry, we did not find any statistically significant differences in MAIT cell frequencies between acute infection compared to convalescence.

We have previously shown that in adults with cholera, peripheral MAIT cells are highly activated at day 7 following infection, and that MAIT cell frequencies remain stable for up to 90 days following infection [14]. Reports of MAIT cell kinetics in the intestinal mucosa are lacking, although studies have shown that MAIT cells are present and active in the gastric mucosa during *H*. *pylori* infection [34], at decreased frequencies in duodenal lamina propria in celiac disease patients compared to healthy controls [31], and decreased in the colon in chronic HIV infection [35, 36]. It was recently reported that MAIT cells are decreased in the circulation and accumulate in the inflamed mucosa of patients with inflammatory bowel diseases, where they display increased cytokine secretion capacities [16, 32, 37, 38]. In addition to this, a decrease in circulating MAIT cell frequencies has been reported previously in studies of live *S*. Typhi [39, 40] and live-attenuated *Shigella dysenteriae* 1 vaccine [7], suggesting that circulating MAIT cells may decrease in frequency in the blood as they move to locally inflamed and infected tissues. In this study, we showed that during cholera, an acute bacterial enteric infection, MAIT cells in the duodenum are activated at levels significantly higher than that in the peripheral blood, based on CD38 expression. Taken together, MAIT cells are present in the lamina propria of the duodenum during cholera, and more activated than those in the blood. We hypothesize that they play an important role in the innate immune response to cholera.

Studies in humans with celiac disease, characterized by increased small bowel permeability, have shown an association between intestinal pathology and loss of intestinal MAIT cells [31]. Similarly, studies of ileal biopsies from patients with inflammatory bowel disease showed an accumulation of MAIT cells in inflamed compared to healthy tissue [16]. We hypothesized that compromised gut barrier function would increase MAIT cell exposure to microbes, resulting in MAIT cell activation and cell death. Thus, we examined two common fecal markers of intestinal inflammation (MPO) and permeability (AAT) [41], and found a high heterogeneity among cholera patients. Notably, we showed that levels of these markers were highly correlated with the loss of duodenal, but not peripheral, MAIT cells. While these findings suggest that MAIT cell loss is associated with intestinal inflammation and permeability, we cannot make conclusions regarding causality or pathogenesis.

Recent studies suggest that variability in MAIT TCR affects microbial ligand discrimination, activation, and phenotype [40, 42, 43]. It has been shown that MAIT cells undergo clonal expansion after *Salmonella enterica* serovar Paratyphi A infection [40]. In our TCR analysis of two donors, we observed that during acute infection (P1) there was a different distribution of MAIT clones in LPLs compared to PBMCs. However, in the convalescent stage (P2), there was overlapping utilization of TCR usage. We hypothesize from these observations that during acute infection, the intestinal compartment may have more layers of functional and phenotypic heterogeneity of MAIT cells than seen in the peripheral blood. The low sample size and lack of paired blood and LPL samples significantly limit our TCR analyses, and thus further studies would be needed to confirm our observations regarding the TCR usage between LPL and blood MAITs, including the significance and generalizability of the TRAJ34 clones isolated.

Our study had a number of limitations. First, we were not able to assess cytokine secretion of MAIT cells during acute and convalescent stages of infection and further investigation of MAIT cell functionality in cholera is warranted. Second, our identification of MAIT cells was based on Vα7.2 antibody, as the MR1-tetramer was not available at the time that this study was conducted (2012–2014). Thus, our results are subject to lack of sensitivity to detect activated MAIT cells, given their potential for down-regulation of CD161 and TCRα chain [44], and may also include a small portion of non-MR1-restricted T cells. Thirdly, our study lacks duodenal biopsy data from healthy (non-cholera) participants, and thus comparisons of MAIT cell activation and frequency were only available between acute and convalescent stages of cholera. Fourthly, our conclusions regarding the immunohistochemical findings are limited by the small sample size and an unblinded operator, and our findings need to be confirmed in larger studies. Lastly, due to ethical and clinical limitations, our sampling of the duodenal mucosa was done only at days 2 and 30. It is likely that MAIT cells, given their innate-like nature in the mucosa, are recruited, activated and release effector molecules early in the course of infection, and that the days examined in our study do not adequately capture the granularity of the kinetics of the MAIT cell response [45–47]. Further studies with more frequent and prolonged sampling would help with examining the kinetics of MAIT cells during *V*. *cholerae* infection.

In conclusion, in this preliminary study limited by small sample size, we have shown that MAIT cells are present in the lamina propria of the duodenum and are highly activated (CD38^+^) compared to peripheral blood during human cholera infection. We hypothesize that the high day 2 MAIT frequency and activation in the duodenum seen in this study reflects the clonal expansion of MAIT cells during the early stages of cholera, though further studies into the functional cytotoxic abilities of duodenal MAIT cells to inactivate *V*. *cholerae* or their roles in innate and adaptive immune responses are needed.

## Supporting information

S1 Table Demographics and vibriocidal antibody titers (to the two V. cholerae O1 serotypes, Ogawa and Inaba) of study subjects. M = male; F = female; D = day. (DOCX)

S2 Table Detailed information for fluorochrome markers used. (DOCX)

S3 Table Number of paired TCRαβ clones found in each sample. PBMC = peripheral blood mononuclear cells; LPL = lamina propria lymphocyte; P1 = Patient 1; P2 = Patient 2; d = day. N = number of times each paired TCRαβ was found.
(DOCX)
