# Supplementary material for: Mucosal-Associated Invariant T (MAIT) cells are highly activated in duodenal tissue of humans with Vibrio cholerae O1 infection: A preliminary report
Source: PLoS Negl Trop Dis. 2022 May 12;16(5):e0010411. doi: 10.1371/journal.pntd.0010411 (PMC9129025; doi:10.1371/journal.pntd.0010411)
Supplement: S1 Table — (DOCX) [file pntd.0010411.s001.docx]

**S1 Table.** Demographics and vibriocidal antibody titers (to the two *V. cholerae* O1 serotypes, Ogawa and Inaba) of study subjects. M = male; F = female; D = day.

|  | |  | |  | |  | **Vibriocidal titer** | | | | | |
| --- | --- | --- | --- | --- | --- | --- | --- | --- | --- | --- | --- | --- |
|  | |  | |  | |  | **Ogawa** | | | **Inaba** | | |
| **Subject ID** | **Gender** | | **Age range** | | **Blood group** | | **D2** | **D7** | **D30** | **D2** | **D7** | **D30** |
| SEGD 17 | M | | 18-30 | | O+ | | 160 | 2560 | 320 | 40 | 1280 | 80 |
| SEGD 18 | M | | 18-30 | | O+ | | 80 | 1280 | 320 | 10 | 80 | 40 |
| SEGD 19 | M | | 31-40 | | B+ | | 160 | 5120 | 1280 | 5 | 40 | 20 |
| SEGD 20 | M | | 31-40 | | O+ | | 5 | 640 | 80 | 5 | 40 | 10 |
| SEGD 21 | M | | 31-40 | | A+ | | 10 | 1280 | 640 | 5 | 40 | 20 |
| SEGD 22 | M | | 18-30 | | A+ | | 5 | 640 | 320 | 5 | 320 | 320 |
| SEGD 23 | M | | 31-40 | | O+ | | 20 | 1280 | 640 | 80 | 640 | 640 |
| SEGD 24 | M | | 18-30 | | A+ | | 40 | 2560 | 1280 | 10 | 1280 | 1280 |
| SEGD 25 | M | | 31-40 | | O+ | | 10 | 2560 | Drop out | 40 | 5120 | Drop out |
| SEGD 26 | F | | 31-40 | | O+ | | 5 | 5120 | 640 | 5 | 2560 | 160 |
| PIC45 | F | | 41-50 | | B+ | | 5 | 5120 | 1280 | 5 | 5120 | 2560 |
| PIC51 | M | | 31-40 | | B+ | | 320 | 5120 | 2560 | 320 | 2560 | 1280 |
| PIC53 | M | | 18-30 | | A+ | | 1280 | 10240 | 1280 | 2560 | 5120 | 2560 |
| PIC94 | M | | 18-30 | | A+ | | 5 | 5120 | 2560 | 80 | 80 | 80 |
| PIC99 | M | | 18-30 | | O+ | | 320 | 10240 | 2560 | 640 | 5120 | 2560 |
