# Supplementary material for: Mucosal-Associated Invariant T (MAIT) cells are highly activated in duodenal tissue of humans with Vibrio cholerae O1 infection: A preliminary report
Source: PLoS Negl Trop Dis. 2022 May 12;16(5):e0010411. doi: 10.1371/journal.pntd.0010411 (PMC9129025; doi:10.1371/journal.pntd.0010411)
Supplement: S2 Table — (DOCX) [file pntd.0010411.s002.docx]

**S2 Table.** Detailed information for fluorochrome markers used.

| **Marker** | **Fluorochrome** | **Clone** | **Company** | **Catalog #** | **Concentration** |
| --- | --- | --- | --- | --- | --- |
| TCR Vα7.2 | PE | 3C10 | Biolegend | 351706 | 1:50 |
| CD3 | PE-TR | 7D6 | Life technologies | MHCD0317 | 1:50 |
| CD4 | Amcyan | SK3 | BD | 339187 | 1:25 |
| CD8 | FITC | RPA-T8 | BD Pharmingen | 561948 | 1:50 |
| CD161 | APC | DX12 | BD Pharmingen | 550968 | 1:25 |
| CD38 | PE-Cy7 | HIT2 | Biolegend | 303516 | 1:50 |
| CD69 | PerCP Cy5.5 | FN50 | BD Pharmingen | 560738 | 1:50 |
| DAPI | - | - | BD Pharmingen | 564907 | 0.1 μg/mL |
