# Supplementary material for: Mucosal-Associated Invariant T (MAIT) cells are highly activated in duodenal tissue of humans with Vibrio cholerae O1 infection: A preliminary report
Source: PLoS Negl Trop Dis. 2022 May 12;16(5):e0010411. doi: 10.1371/journal.pntd.0010411 (PMC9129025; doi:10.1371/journal.pntd.0010411)
Supplement: S3 Table — PBMC = peripheral blood mononuclear cells; LPL = lamina propria lymphocyte; P1 = Patient 1; P2 = Patient 2; d = day. N = number of times each paired TCRαβ was found. (DOCX) [file pntd.0010411.s003.docx]

**S3 Table.** Number of paired TCRαβ clones found in each sample. PBMC = peripheral blood mononuclear cells; LPL = lamina propria lymphocyte; P1 = Patient 1; P2 = Patient 2; d = day. N = number of times each paired TCRαβ was found.

| Sample | TCR | N |
| --- | --- | --- |
| P1[PBMC] d2 | TRAV1-2 TRAJ33 TRBV7-3 TRBJ2-2 | 1 |
|  | TRAV1-2 TRAJ6 TRBV7-3 TRBJ2-5 | 1 |
|  | TRAV1-2 TRAJ34 TRBV7-9 TRBJ2-7 | 1 |
|  | TRAV1-2 TRAJ8 TRBV30 TRBJ2-2 | 1 |
| P1[LPL] d2 | TRAV1-2 TRAJ33 TRBV7-2 TRBJ2-2 | 1 |
| P1[PBMC] d7 | TRAV1-2 TRAJ33 TRBV20-1 TRBJ1-1 | 1 |
|  | TRAV1-2 TRAJ33 TRBV20-1 TRBJ2-7 | 1 |
|  | TRAV1-2 TRAJ34 TRBV7-2 TRBJ2-2 | 3 |
|  | TRAV1-2 TRAJ33 TRBV7-2 TRBJ2-3 | 1 |
|  | TRAV1-2 TRAJ34 TRBV7-3 TRBJ2-2 | 1 |
| P2[PBMC] d180 | TRAV1-2 TRAJ33 TRBV6-6 TRBJ2-5 | 1 |
|  | TRAV1-2 TRAJ33 TRBV6-4 TRBJ2-3 | 2 |
|  | TRAV1-2 TRAJ33 TRBV20-1 TRBJ2-2 | 3 |
|  | TRAV1-2 TRAJ13 TRBV7-2 TRBJ2-2 | 2 |
|  | TRAV1-2 TRAJ33 TRBV7-2 TRBJ2-2 | 4 |
|  | TRAV1-2 TRAJ33 TRBV4-2 TRBJ2-1 | 1 |
|  | TRAV1-2 TRAJ13 TRBV20-1 TRBJ2-2 | 1 |
|  | TRAV1-2 TRAJ33 TRBV20-1 TRBJ2-3 | 1 |
|  | TRAV1-2 TRAJ33 TRBV30 TRBJ2-2 | 1 |
|  | TRAV1-2 TRAJ34 TRBV4-2 TRBJ2-1 | 1 |
| P2[LPL] d180 | TRAV1-2 TRAJ34 TRBV7-2 TRBJ2-2 | 2 |
|  | TRAV1-2 TRAJ33 TRBV6-4 TRBJ2-2 | 1 |
|  | TRAV1-2 TRAJ26 TRBV30 TRBJ2-2 | 1 |
|  | TRAV1-2 TRAJ33 TRBV7-2 TRBJ2-2 | 3 |
|  | TRAV1-2 TRAJ33 TRBV6-2 TRBJ2-7 | 1 |
|  | TRAV1-2 TRAJ20 TRBV6-1 TRBJ2-1 | 1 |
|  | TRAV1-2 TRAJ33 TRBV20-1 TRBJ1-2 | 1 |
|  | TRAV1-2 TRAJ13 TRBV7-2 TRBJ2-2 | 4 |
|  | TRAV1-2 TRAJ27 TRBV4-2 TRBJ2-1 | 1 |
|  | TRAV1-2 TRAJ33 TRBV4-2 TRBJ2-1 | 1 |
|  | TRAV1-2 TRAJ33 TRBV6-4 TRBJ2-3 | 1 |
|  | TRAV1-2 TRAJ33 TRBV6-1 TRBJ2-1 | 1 |
|  | TRAV1-2 TRAJ33 TRBV30 TRBJ2-2 | 1 |
