# Supplementary material for: Mucosal-Associated Invariant T (MAIT) cells are highly activated in duodenal tissue of humans with Vibrio cholerae O1 infection: A preliminary report
Source: PLoS Negl Trop Dis. 2022 May 12;16(5):e0010411. doi: 10.1371/journal.pntd.0010411 (PMC9129025; doi:10.1371/journal.pntd.0010411)
Supplement: Attachment — Submitted filename: pNTD 21-00600R1 Response to reviewers.pdf [file pntd.0010411.s005.pdf]

March 15, 2022

Khitam Muhsen
Guest Editor
PLOS Neglected Tropical Diseases

Alfredo Torres
Deputy Editor
PLOS Neglected Tropical Diseases

Subject: Revised version of manuscript **PNTD-D-21-00600R1**

Dear Drs. Muhsen and Torres,

Please find attached our revised manuscript "Mucosal-Associated Invariant T (MAIT) Cells are Highly Activated in Duodenal Tissue of Humans with Vibrio cholerae O1 Infection." We have revised the manuscript based on the comments received from reviewers, with changes highlighted in yellow. Detailed responses to each comment are as below (in dark blue). Lines referred to are in the "tracked changes" document.

Given the sample size in this study, the preliminary nature of the data it is recommended to add "a preliminary report" to the title. In addition, more details on the sample size, age and sex of the patients should be added to the abstract and summary.

In the conclusions (both abstract / discussion) please highlight the small sample size. Please also moderate the conclusions, (especially when some results were not statistically significant at $p<0.05$) and highlight the preliminary nature of the report.

Response: Thank you for your suggestions. We have inserted the participants numbers, median and range of age, and sex, in the abstract and summary. We have highlighted the small sample size and the preliminary nature of the report in the conclusions of the abstract and discussion. We have revised the article title as suggested.

Figures legend – please enter the number of samples tested in each group/time points. Figure 2: When using ANOVA please check if the underling assumptions of ANOVA were fulfilled, otherwise please use non-parametric test.

Response: Figure legends now include number of samples tested. For Fig 2, we have confirmed that the data showed that assumptions of ANOVA were fulfilled (passed normality tests of Anderson-Darling and Shapiro-Wilk).

****

Reviewer's Responses to Questions

Methods

Reviewer #1: (No Response)

Reviewer #2: small sample size, but noting these samples are difficult to obtain and this is highlighted in the manuscript.

Reviewer #3: The weaknesses of the study have now been better spelled out in the manuscript.

Results

Reviewer #1: (No Response)

Reviewer #2: (No Response)

Reviewer #3: The inconsistencies in the result section have been clarified in the revised version of the manuscript. However, immunofluorescence pictures are not of an acceptable quality.

Response: We have replaced the representative image in Figure 1A with another portion of the scanned slide that has better views of the crypt and villi structures, and positioned the arrows at more prominent MAIT cells.

Conclusions

Reviewer #1: (No Response)

Reviewer #2: see comments below

Reviewer #3: These issues are adequately addressed in the revised manuscript.

Editorial and Data Presentation Modifications

Reviewer #1: (No Response)

Reviewer #2: see comments below

Reviewer #3: (No Response)

Summary and General Comments

Reviewer #1: All the points raised by this reviewer have been addressed satisfactorily.

Reviewer #2: The authors have revised their manuscript addressing most comments resulting in an improved manuscript. They have removed some less convincing data (previous figure 4). Some issues remain as listed below.

Previous comment. 1. The histology images shown in Fig 1A is of low resolution (even in downloaded.tif file). Some of the arrows for Va7.2 staining do not appear to be pointing to cells. It is hard to assess from the histology presented if statements about location in the LP are correct (line 183).

Response: Thank you for your suggestion. Unfortunately, this is the highest resolution and magnification we were able to obtain. These images were obtained and saved with this resolution in 2013, and we are unable to improve the resolution of the saved images.

Remaining issues: the response does not address the main issues. It is still difficult to determine the validity of data presented in B and C when some arrows do not appear to be pointing to cells. Crypt and villi structures are hard to see. Noting the difficulty in sourcing these samples the authors could at least list this as a limitation and ensure their conclusions based on these results are appropriately phrased.

Response: We have replaced the representative image in Figure 1A with another portion of the scanned slide that has better views of the crypt and villi structures, and positioned the arrows at more prominent MAIT cells.

Previous comment. 2. How was the quantitation done in Fig 1B and C? i.e. how many sections, from how many biopsies were enumerated for each patient? Was the operator blinded? What is meant by “paired duodenal biopsies” in line 181?

Response: We have added to the Methods section (lines 145-147), “For each patient, a single operator, using ImageJ software, enumerated both MAIT and CD3+ cells from a single biopsy section at each time point from paired (available at both day 2 and day 30) samples.”

Remaining issues: The response does not fully address the previous comment. Was the operator blinded to the time points for each biopsy?

What were the absolute numbers counted? Were more biopsies available? What was the variation in numbers between patients? Drawing conclusions from such small numbers of biopsies is problematic in this reviewer’s opinion, particularly if the operator was not blinded to the grouping.

Response: We have clarified that the operator was not blinded during the quantification of each section (line 147), and that it was from a single biopsy section at each time point (line 148). We have removed from the abstract the immunohistochemistry findings of the frequencies of MAIT cells, removed the sentences in the Discussion discussing the difference in findings (lines 294-303), and replaced with statement (lines 292-294), "Using immunohistochemistry and flow cytometry, we did not find any statistically significant differences in MAIT cell frequencies between acute infection compared to convalescence." We have added to the limitations paragraph (lines 360-363), "Fourthly, our conclusions regarding the immunohistochemical findings are limited by the small sample size and an unblinded operator, and our findings need to be confirmed in larger studies."

Previous comment. 3. How do the authors explain the inconsistency in terms of %MAITs of CD3+ T cells in duodenal samples between data shown in Fig 1B vs Fig 2A and Fig 1C vs 2B? In Fig. 1B, authors claimed that frequency of MAIT cells was significantly higher at D2 compare to D30. However, no difference was observed in Fig 2A.

Response: To address the discrepancy between flow cytometry and immunohistochemistry, we have now added to the discussion (line 291-298) as follows, "A potential explanation for this discrepancy include differences in sample preparation for flow cytometry and immunohistochemistry, as embedding and fixation of some antigenic epitopes could be lost during the processing and preparation of specimens, and collagenase treatment used to prepare samples for flow cytometry can also impact expression of surface markers [36, 37]. Furthermore, there is a lower detection threshold of flow cytometry compared with immunohistochemistry, whereby dim antigen expression may be easily distinguished from background. Such discrepancies between flow cytometric and immunohistochemical results have been reported by others [38-40]."

Remaining issues: It is unclear how these factors would differentially affect the results at day 2 and day 30.

Additionally, the histology differences are no longer reported as significant ($p = 0.06$) as the authors have changed the statistical test used, and thus claims of differences between the two time points should be avoided.

Response: As above, we have removed from the abstract the immunohistochemistry findings of the frequencies of MAIT cells, removed the sentences in the Discussion discussing the difference in findings (lines 294-303), and replaced with statement (lines 292-294), "Using immunohistochemistry and flow cytometry, we did not find any statistically significant differences in MAIT cell frequencies between acute infection compared to convalescence."

Previous comment. 4. It is a worry that the authors claim to show MAIT cell frequencies increase in acute infection vs convalescence (line 282) based on histology data, but that they "were unable to replicate this finding using flow cytometric analysis...". This is cherry-picking since they could equally state there was no difference based on the flow data. Given that neither assessment used MR1 tetramers, which would precisely detect MAIT cells, can the authors be sure that the markers used do not

change expression during activation leading to loss of signal and apparent decrease in MAIT cells?

Response: Unfortunately, during the period of this study (2012-2014), the MR1-tetramer was not available. Thus, we initially identified MAIT as CD3+CD4−CD161hiVα7.2+ for flow cytometry as we have used in our earlier publication (Ref 14). For immunohistochemistry, we used the staining markers for MAIT cells published previously by other groups (PMID 21084709, 24450998).

We have now added this statement to the Methods section (line 136-138): "In line with immunohistochemical methods from reports available at time of the experiments [16, 17]," To be more consistent with immunohistochemistry methods, we have revised our identification of MAIT cells by flow to not include CD4 gating (i.e. MAITs are CD3+CD161hiVα7.2+ cells), and we have made this changed in Methods section (line 129). We have now highlighted the discrepancy between flow cytometry and immunohistochemistry findings in the Discussion, as quoted in response to the question above.

Remaining issues: The flow cytometry data does not appear to be changed (Fig 2) to reflect the change in gating. As above, claims of "differences" should be avoided when not significant.

Response: We apologize for this confusion, the flow data in figure 2 submitted originally and in revised manuscript was gated on CD3+ cells as shown in Fig 2E gating strategy. We had made an error in the text that has been corrected in the revision (line 132).

Previous comment. 5. In flow cytometry data, MAIT cells are defined as CD3+CD4−CD161hiVα7.2+ cells. However, some MAIT cells express CD4. It would be interesting to look at CD4 (and CD8) coreceptor expression if this data is available.

Response: Thank you for pointing this out. We have examined the flow cytometry data with and without CD4 involved in the gating strategy and this did not change any of our findings (no differences between day 2 and day 30). To address comments above and also posed by other reviewers, to be more consistent with MAIT identification by immunohistochemistry, we have changed the definition of MAIT cells to not include CD4 (as noted in statement above). Unfortunately, at the time of this study, we did not stain cells with CD8 antibody.

Remaining issues: OK. But data in figures does not appear to be changed.

Response: As with response above, the data were always shown gated on CD3+ T cells. We had made an error in the text that has been corrected in the revision.

Previous comment. 9. For Fig. 4, FACS plots showing β7 expression on peripheral MAIT and non-MAIT cells should be included. B7 data from healthy control is also required.

Response: Given the lack of integrin β7 data from controls, and the limited information gained, we have decided to remove these data from our manuscript.

Remaining issues (minor): Integrin B7 Ab still appears in methods (line 123).

Response: Integrin Beta 7 is now deleted from the methods (line 126).

Previous comment. 21. The data in Fig 5E doesn't make sense. Frequencies should add up to 100 (line 254- 259 in text).

Response: We apologize for this confusing figure. The Y axis is the absolute number of times (not percentage) of CDR3b with a particular amino acid length. This has been corrected in results (line 263 is updated to number instead of frequency), the y-axis title, and the figure legend (line 429) has been modified to, “x-axis shows length distribution of amino acids and y axis shows number of times CDR3β sequence was found with that amino acid length.”

Remaining issues: The figure now makes sense. However, if the authors wish to compare LPL to PBMC CDR3b length (line 263-) they need to consider the percentage of sequences rather than the numbers with each length. It seems the numbers in LPL at d2 are lower than PBMC due to the total number of sequences rather than a different pattern of CDR3b length. In fact, according to the results in Fig 5E (new 4E), a similar pattern of CDR3β sequence seems to be observed in MAIT cells from LPL and PBMC, with most frequent 14 nucleotide length.

Response: We thank the reviewer for this suggestion. We have now corrected the figure and figure legend with y-axis showing “percentage of CDR3b sequences” as requested. Text is updated as follows (lines 266-269), “We observed that the percentage frequency of CDR3β sequences with 14 nucleotides was highest, and relatively higher in LPL compared to PBMCs. In contrast, the percentage of CDR3β sequences with 15 nucleotides was relatively higher in PBMCs compared to LPL samples (Figure 4E).”

Reviewer #3: The revised manuscript is much improved, and many inconsistencies have been addressed or clarified.

However, the resolution of the imunoflourescence pictures is still very poor, and they can’t be used to verify the conclusions made. The authors argue that the experiments were performed in 2013, and that the quality can’t be improved. If so, I would suggest that another set of biopsies are collected and evaluated using equipment that will yield pictures of sufficient quality to confirm the conclusions made previously.

Likewise, it is argued that as the experiments were performed before the MR1 tetramers became available, the authors can’t confirm the MR1 reactivity of the cells identified as MAIT cells. I would suggest a round of new FACS analyses using biopsies from cholera-infected or convalescent patients to confirm that the CD161+Va7.2+ cells detected in the duodenal mucosa are actually MR1-reactive.

Response: Thank you for your suggestions. We have replaced the representative image in Figure 1A with another portion of the scanned slide that has better views of the crypt and villi structures, and positioned the arrows at more prominent MAIT cells. Regarding MR1-tetramer reactivity, unfortunately, due to shipping delays and shipping price increases during this pandemic, and running out of funding, we have not been able to ship necessary reagents to Bangladesh to perform the suggested experiment in cholera patients. However, others have already shown the MR1 reactivity of CD161+Va7.2+ cells in human healthy jejunum (Reantragoon et al, *J Exp Med*, 2013) and human gastric mucosa during infection (Ming S, et al, *Front Immunol*, 2021), and our group has recently also shown this in another type of mucosal tissue (tonsils, Jensen O, et al, *Sci Immunology*, 2022), and we have in the Discussion stated the limitation (Lines 355-358), “Thus, our results are subject to lack of sensitivity to detect activated MAIT cells,

given their potential for down-regulation of CD161 and TCR alpha chain [51], and may also include a small portion of non-MR1-restricted T cells."

We hope we have been able to satisfactorily respond to the comments raised by the reviewers and that the paper is now suitable for acceptance in PLOS Neglected Tropical Diseases.

Thank you again for your consideration of this manuscript.

Sincerely yours,

Daniel T. Leung, MD, MSc, FASTMH, FIDSA
Corresponding author
Associate Professor
Division of Infectious Diseases
University of Utah School of Medicine
30 N 1900 E, RM 4C416
Salt Lake City, UT, USA
Email: Daniel.Leung@utah.edu
