# Supplementary material for: Mucosal-Associated Invariant T (MAIT) cells are highly activated in duodenal tissue of humans with Vibrio cholerae O1 infection: A preliminary report
Source: PLoS Negl Trop Dis. 2022 May 12;16(5):e0010411. doi: 10.1371/journal.pntd.0010411 (PMC9129025; doi:10.1371/journal.pntd.0010411)
Supplement: Attachment — Submitted filename: pNTD 21-00600 Response to reviewers.docx [file pntd.0010411.s004.docx]

September 17, 2021

Khitam Muhsen

Guest Editor

PLOS Neglected Tropical Diseases

Alfredo Torres

Deputy Editor

PLOS Neglected Tropical Diseases

Subject: Revised version of manuscript **PNTD-D-21-00600**

Dear Drs. Muhsen and Torres,

Please find attached our revised manuscript “Mucosal-Associated Invariant T (MAIT) Cells are Highly Activated in Duodenal Tissue of Humans with Vibrio cholerae O1 Infection.” We have revised the manuscript based on the comments received from reviewers, with changes highlighted in yellow. Detailed responses to each comment are as below (in dark blue).

--------------------

Methods

Reviewer #1: Please see "Summary and General Comments" section

Reviewer #2: The population is not fully described, healthy controls are lacking and the sample sizes are small in some analyses. See full comments below.

Reviewer #3: The objectives are reasonably outlined, and the study design appropriate.

The population is well described and also appropriate for the study. The main worry here is the population size, as the inter-individual variation is large. The number of study patients is low, which makes it hard to make solid conclusions.

It is unclear from the text which statistical methods have actually been used in Fig. 1 and Fig. 4, the M&M section states Wilcoxon and the figure legends t-test.

No ethical concerns.

Response: See comments addressed below.

--------------------

Results

Reviewer #1: Please see "Summary and General Comments" section

Reviewer #2: There are unexplained inconsistencies between the data presented in different figures.

See full comments below.

Reviewer #3: The analysis is according to plan.

There are some issues with regard to result presentation. The IF staining in Fig. 1A is hard to interpret. Please provide a close up of the indicated MAIT cells, to show how they can be distinguished from the background. Please also show the DAPI stain so that individual cells can be identified.

There is also a lack of FMO controls in Fig. 2, which would be needed to judge the cut off for positive CD38 and CD39 staining.

Furthermore, the labeling of the axes in the dot plots in Fig. 2 and of the samples in Fig. 5A-D is very hard to read and clarity can be improved.

Response: See comments addressed below.

--------------------

Conclusions

Reviewer #1: Please see "Summary and General Comments" section

Reviewer #2: Conclusions are not always fully supported. See full comments below.

Reviewer #3: The low patient number makes it hard to draw solid conclusions. This limitation in mentioned, but not elaborated. The conflicting results from IHC and flow cytometry and how data can be used to advance understanding of the topic are not really discussed either.

Response: See comments addressed below.

--------------------

Editorial and Data Presentation Modifications?

Reviewer #1: (No Response)

Reviewer #2: Several small modifications. See fully comments (under minor comments heading) below.

Reviewer #3: 1. The first three references are quite old, and there are more recent reviews on the topic that could be used to introduce the reader to the topic of MAIT cells.

Response: We have now replaced these references with more recent reviews

2. The first paragraph of the result section would be better suited in the materials and methods. Please also define VE blood group antigens in Suppl. Table 1.

Response: We have now moved the first paragraph of the Results section to the Methods section (Lines 97-103). We have also clarified “+VE” with “+” in Suppl Table 1.

3. The M&M sections states that Wilcoxon was used to compare values before and after infection, but in the legends to Fig. 1 and 4 it say paired t-test. Please clarify.

Response: We have consulted with our statistician, who recommends that the Wilcoxon ranked sum test be used for analyses in Figure 1, given the concern for violating normality assumption with only 5 paired observations. We have now clarified this in Methods, and Figure Legends. Given that we have now moved to a non-parametric test, the statistical significance is lost, as p = 0.06. We have changed Results (lines 195-197) to, “MAIT cells, as a % of total CD3+ cells, was statistically non-significantly higher at day 2 of infection compared to day 30 (p = 0.06, Figure 1B), though this was the smallest achievable p-value with only 5 paired observations.”

4. In Fig. 1C, only 1-4% of lamina propria lymphocytes are CD3+. This is surprisingly low, has there maybe been a mistake in the labeling of the y-axis?

Response: We apologize for the mislabeling – this was the % of CD3+ in total lamina propria area (not of total LP lymphocytes). We have now revised the axis in Figure 1C.

5. In the text (line 193 and 216) it says that 7 patients were available for biopsies day 30, but in the corresponding plot in Fig. 2A, 8 individuals are shown. Please clarify.

Response: You are correct, sorry about the typo, we have revised to 8 individuals.

6. In M&M, it is stated that cells were sorted from one donor for TCR analysis (line 146) while in the result section, data from two individuals are presented (line 237). Please clarify. The second patient appears to be biopsied at day 180, which is not mentioned in the M&M. Furthermore, how were these patients selected?

Response: We apologize for this error, and have now clarified in the Methods section (line 162-163), “MAIT cells from PBMCs and LPLs from one donor, at acute stages of infection (days 2 and 7) and from a second donor at a convalescent stage of infection (day 180).” These patients were part of the convenience sample enrolled for this study, under the inclusion/exclusion criteria defined in Methods section, and were chosen for TCR analysis based on availability of TCR sequencing reagents at time of enrollment.

7. Reference 32 and 33 are the same.

Response: Duplicate references has now been removed.

--------------------

Summary and General Comments

**Reviewer #1:** The study by Bhuiyan et al. characterized MAIT cells present in duodenal biopsies and their paired peripheral blood samples in patients with culture-confirmed Vibrio cholerae O1 infection. The manuscript is sound, interesting, and presents novel findings reported straightforwardly.

A few suggestions are below:

1. Please transfer the paragraph containing the demographics from the “Results” (lines 171-177) to the “methods” section (i.e., “Study population and sample collection” section) of the manuscript.

Response: Information containing the demographics have been transferred to the methods section as suggested (line 97-103).

2. Please explain, in the manuscript, the rationale for identifying MAIT cells using different markers (e.g., CD161 for blood and IL-18Rα for duodenal biopsies). The readers may be confused by a choice that is not explained.

Response: During the period of this study (2012-2014), the MR1-tetramer was not available. Thus, we identified MAIT as CD3^+^CD4^-^CD161^hi^Vα7.2^+^ for flow cytometry as we have used in our earlier publication (Ref 14). For immunohistochemistry, we used the staining markers for MAIT cells published previously by other groups (PMID 21084709, 24450998). It was not until 2019 and with a chip cytometry protocol that the use of CD161 was reported in evaluations of MAIT cells (PMID 31533032). We have now added this statement to the Methods section (line 136-138): “In line with immunohistochemical methods from reports available at time of the experiments [16, 17]…”

3. Figure 1. Panel A would benefit from a figure with higher resolution and magnification.

Response: Thank you for your suggestion. Unfortunately, this is the highest resolution and magnification we were able to obtain. These images were obtained and saved with this resolution in 2013, and we are unable to improve the resolution of the saved images.

4. Please confirm that day 2 and 30 biopsies were collected from the same area of the duodenum and not randomly. Add a statement clarifying this issue into the manuscript. Also, discuss further the discrepancy between duodenum MAIT cell frequencies obtained by immunochemistry (Fig. 1B) and those obtained by flow cytometry (Fig. 2A). Is it a sampling issue? Or experiment artifact? Collagenase might have impacted the expression of MAIT specific markers... Further immunochemistry stainings are necessary to clarify this issue.

Response: Duodenal biopsies were taken from the second part of the duodenum from every patient. We have now stated in the methods section (line 94-95) that “Using standard forceps, we obtained approximately six duodenal pinch biopsies (from second part of duodenum) of approximately 1 mm^3^ in diameter from each patient at each time point.”

Regarding the discrepancy between flow cytometry and immunohistochemistry, we have now added to the discussion (line 291-298) as follows “A potential explanation for this discrepancy include differences in sample preparation for flow cytometry and immunohistochemistry, as embedding and fixation of some antigenic epitopes could be lost during the processing and preparation of specimens, and collagenase treatment used to prepare samples for flow cytometry can also impact expression of surface markers [36, 37]. Furthermore, there is a lower detection threshold of flow cytometry compared with immunohistochemistry, whereby dim antigen expression may be easily distinguished from background. Such discrepancies between flow cytometric and immunohistochemical results have been reported by others [38-40].”

5. References 38 and 39 are the same.

Response: The duplicate reference has now been removed.

6. The discussion lacks publications showing MAIT cell kinetics (e.g., PMID: 23898209, PMID: 32811991, PMID: 28428786). Given that the authors dwell on the effects of MAIT cells in the duodenal biopsies and blood, it would perhaps be helpful if, at the end of the discussion, they committed to a statement on the pattern of the MAIT cell kinetics and its implications on cholera pathology.

Response: We have now added the following to the Discussion (line 355-362), “Lastly, due to ethical and clinical limitations, our sampling of the duodenal mucosa was done only at days 2 and 30. It is likely that MAIT cells, given their innate-like nature in the mucosa, are recruited, activated and release effector molecules early in the course of infection, and that the days examined in our study do not adequately capture the granularity of the kinetics of the MAIT cell response [52-54]. Further studies with more frequent and prolonged sampling would help with examining the kinetics of MAIT cells during V. cholerae infection,” and in Lines 366-368, “We hypothesize that the high day 2 MAIT frequency and activation in the duodenum seen in this study reflects the clonal expansion of MAIT cells during the early stages of cholera…”

7. Since no kinetic changes occurred in either blood or duodenum, please discuss the need for extra time points to establish the impact of MAIT cells on cholera infection

Response: We have now added the following to the Discussion (line 360-362), “Further studies with more frequent and prolonged sampling would help with examining the kinetics of MAIT cells during V. cholerae infection.”

8. Material & Methods. There is no statement about blood collection at days 7 and 180, biopsies at day 180, or integrin β7 staining. If an oversight, please fix it.

Response: We have now added more details regarding blood and duodenal biopsy collection in the Methods section as suggested (lines 91-97), “After informed consent, we performed esophagogastroduodenoscopy (EGD) on day 2 (acute illness phase), day 30 (convalescent phase), and day 180 (late convalescent phase) following admission. Using standard forceps, we obtained approximately six duodenal pinch biopsies (from second part of duodenum) of approximately 1 mm^3^ in diameter from each patient at each time point. We also obtained a stool sample from each patient at day 2 and venous blood samples at days 2, 7, 30 and 180.” Given reviewer comments, including the lack of data from controls, and the limited information gained, we have decided to remove the integrin β7 results from our manuscript.

9. Fix typo. “Results” section states, “The majority of MAIT cells were identified in the lamina propria, with no cells identified in the villous and crypt epithelia (lines 183 & 184), but the discussion describes, “we demonstrated that the vast majority of MAIT cells are in the lamina propria, predominantly in the crypts, and rarely in the epithelia.”( lines 271-273). In the discussion, do you imply beneath the crypts?

Response: We have fixed the typo in results (lines 193-194), “The majority of MAIT cells were identified in the lamina propria of the crypt, with no cells identified in the villi,” and discussion (line 279-281): “we demonstrated that the vast majority of MAIT cells are in the lamina propria, predominantly in the crypt, and rarely in the villi.”

**Reviewer #2:** Summary

MAIT cells are known to be activated by diverse microbes and several mouse studies have shown their role in protecting against bacterial infection, but also in driving pathology in chronic infection. Studies of MAIT cells in humans during infection, particularly at mucosal sites, are currently limited.

Here, Bhuiyan and colleagues assess MAIT cell numbers in acute and convalescent Vibrio cholerae infection in duodenal biopsies and bloods from a small cohort of patients. The topic is interesting and the study would potentially add important information to the field. However, much of the data presented seems preliminary (for example the clonal expansion observed in one patient). He study lacks healthy control samples (which could easily be included for PBMCs if not biopsies). There is a relatively small number of patient used, and only small subsets of these analysed for the data shown in different figures (5 patients for some).

There are also inconsistencies between data from flow cytometry and histology that are not explained, and these then also affect downstream analyses.

Detailed comments below offer suggestions to improve the study.

Major comments

1. The histology images shown in Fig 1A is of low resolution (even in downloaded.tif file). Some of the arrows for Va7.2 staining do not appear to be pointing to cells. It is hard to assess from the histology presented if statements about location in the LP are correct (line 183).

Response: Thank you for your suggestion. Unfortunately, this is the highest resolution and magnification we were able to obtain. These images were obtained and saved with this resolution in 2013, and we are unable to improve the resolution of the saved images.

2. How was the quantitation done in Fig 1B and C? i.e. how many sections, from how many biopsies were enumerated for each patient? Was the operator blinded? What is meant by “paired duodenal biopsies” in line 181?

Response: We have added to the Methods section (lines 145-147), “For each patient, a single operator, using ImageJ software, enumerated both MAIT and CD3+ cells from a single biopsy section at each time point from paired (available at both day 2 and day 30) samples.”

3. How do the authors explain the inconsistency in terms of %MAITs of CD3+ T cells in duodenal samples between data shown in Fig 1B vs Fig 2A and Fig 1C vs 2B? In Fig. 1B, authors claimed that frequency of MAIT cells was significantly higher at D2 compare to D30. However, no difference was observed in Fig 2A.

Response: To address the discrepancy between flow cytometry and immunohistochemistry, we have now added to the discussion (line 291-298) as follows, “A potential explanation for this discrepancy include differences in sample preparation for flow cytometry and immunohistochemistry, as embedding and fixation of some antigenic epitopes could be lost during the processing and preparation of specimens, and collagenase treatment used to prepare samples for flow cytometry can also impact expression of surface markers [36, 37]. Furthermore, there is a lower detection threshold of flow cytometry compared with immunohistochemistry, whereby dim antigen expression may be easily distinguished from background. Such discrepancies between flow cytometric and immunohistochemical results have been reported by others [38-40].”

4. It is a worry that the authors claim to show MAIT cell frequencies increase in acute infection vs convalescence (line 282) based on histology data, but that they “were unable to replicate this finding using flow cytometric analysis…”. This is cherry-picking since they could equally state there was no difference based on the flow data. Given that neither assessment used MR1 tetramers, which would precisely detect MAIT cells, can the authors be sure that the markers used do not change expression during activation leading to loss of signal and apparent decrease in MAIT cells?

Response: Unfortunately, during the period of this study (2012-2014), the MR1-tetramer was not available. Thus, we initially identified MAIT as CD3^+^CD4^−^CD161^hi^Vα7.2^+^ for flow cytometry as we have used in our earlier publication (Ref 14). For immunohistochemistry, we used the staining markers for MAIT cells published previously by other groups (PMID 21084709, 24450998).

We have now added this statement to the Methods section (line 136-138): “In line with immunohistochemical methods from reports available at time of the experiments [16, 17],” To be more consistent with immunohistochemistry methods, we have revised our identification of MAIT cells by flow to not include CD4 gating (i.e. MAITs are CD3^+^CD161^hi^Vα7.2^+^ cells), and we have made this changed in Methods section (line 129). We have now highlighted the discrepancy between flow cytometry and immunohistochemistry findings in the Discussion, as quoted in response to the question above.

Regarding the limitation of the lack of MR1 tetramer use, we have added to the Discussion (lines 348-353), “our identification of MAIT cells was based on Va7.2 antibody, as the MR1-tetramer was not available at the time that this study was conducted (2012-2014). Thus, our results are subject to lack of sensitivity to detect activated MAIT cells, given their potential for down-regulation of CD161 and TCR alpha chain [51], and may also include a small portion of non-MR1-restricted T cells.” We are carrying out further in-depth MAIT analyses with MR1 tetramers in our current work and hope we will able to report those data in a future report.

5. In flow cytometry data, MAIT cells are defined as CD3+CD4−CD161hiVα7.2+ cells. However, some MAIT cells express CD4. It would be interesting to look at CD4 (and CD8) coreceptor expression if this data is available.

Response: Thank you for pointing this out. We have examined the flow cytometry data with and without CD4 involved in the gating strategy and this did not change any of our findings (no differences between day 2 and day 30). To address comments above and also posed by other reviewers, to be more consistent with MAIT identification by immunohistochemistry, we have changed the definition of MAIT cells to not include CD4 (as noted in statement above). Unfortunately, at the time of this study, we did not stain cells with CD8 antibody.

6. What is the percentage of MAIT cells among CD3+ cell in cholera-free duodenal tissue? Without such data, it is hard to claim that “MAIT cell frequencies are significantly increased during acute infection compared to convalescence”.

Response: Unfortunately, for this study, we did not have ethical approval to take biopsies from cholera-free participants. We have added this limitation in the discussion (lines 439-441), “our study lacks duodenal biopsy data from healthy (non-cholera) participants, and thus comparisons of MAIT cell activation and frequency were only available between acute and convalescent stages of cholera.”

7. For Fig. 2C and D, duodenal MAIT cells highly express CD38 and CD69 at both acute and convalescent stages of cholera. Is this an intrinsic property of duodenal (or tissue) MAIT cells or induced by bacterial infection? In particular, CD69 expression is known to be high in tissues compared to PBMCs. In order to claim that duodenal MAIT cells in cholera infection are activated based on these markers (line 137), it is necessary to examine CD38 and CD69 expression on MAIT cells from uninfected duodenal samples.

Response: Unfortunately, for this study, we did not have ethical approval to take biopsies from cholera-free participants. We have added this limitation in the discussion (lines 353-355), “Thirdly, our study lacks duodenal biopsy data from healthy (non-cholera) participants, and thus comparisons of MAIT cell activation and frequency were only available between acute and convalescent stages of cholera.”

8. For Fig. 2E, isotype or FMO control should be shown for CD69 and CD38 staining

Response: We have now added FMO controls to new Fig. 2E.

9. For Fig. 4, FACS plots showing β7 expression on peripheral MAIT and non-MAIT cells should be included. Β7 data from healthy control is also required.

Response: Given the lack of integrin β7 data from controls, and the limited information gained, we have decided to remove these data from our manuscript.

10. For Fig. 5A, is the dominant MAIT cell clone in the LPL a result of selective proliferation? Or preferential tissue homing? Again, uninfected controls would be welcomed here.

Response: Based on the available data generated, it is difficult to conclude if any of the clones in the LPL is a result of selective proliferation or preferential tissue homing. We have now added to the Results section (lines 250-254): “Unfortunately, despite many unpaired TCRα and TCRβ sequences available from the acute day 2 LPL sample (Figure 4A-C), there was only one paired TCRαβ clone sequenced (Supplemental Table 2), and thus comparisons between LPL and PBMC clonality could not be made based on paired TCR reads. On the other hand, numerous LPL paired TCR reads were successfully resulted for the convalescent stage (day 180) donor, for whom PBMC (89 cells) and LPL (91 cells) were sequenced, and we found overlapping MAIT TCR repertoire (Figure 4D and Table S1), with the majority of PBMC and LPL MAIT cells expressed TRAJ33 (Figure 4A), TRBV7-2, TRBV20-1 or TRBV30 (Figure 4B) and TRBJ 2-2 (Figure 4C).” Unfortunately, we do not have ethical permission to sample from uninfected controls, and thus we have also added to the limitations section (lines 353-355), “Thirdly, our study lacks duodenal biopsy data from healthy (non-cholera) participants, and thus comparisons of MAIT cell activation and frequency were only available between acute and convalescent stages of cholera.”

Minor comments

11. Line 60. “The ligand for MAIT cells…” This should be “antigen” and there is more than one described.

Response: We have revised accordingly (line 56).

12. TNFa should be TNF consistent with current naming convention.

Response: We have revised in the manuscript (line 61) as suggested.

13. The methods are light on detail and insufficient for others to repeat. For example, antibodies should be listed with clone name, catalogue numbers and concentrations used for staining.

Response: We have added a supplemental table 3 with these details.

14. The patient information should be more detailed. For example, Inaba and Ogawa are only mentioned in Supp Table 1 and not explained. Are these patients part of a larger study? What are SEGD and PIC in the patient numbers? Which patient samples were used for FACS vs histology? The antibiotics given should be described along with the timing relative to biopsies (line 94). Is the use of these antibiotics known or anticipated to affect the MAIT cell response?

Response: Thank you for these suggestions. The antibiotics were given 18-24 hours prior to study enrolment are not known to nor anticipated to affect the MAIT response. We have now provided additional information in the manuscript regarding antibiotics (line 90), “….provided antibiotics per hospital protocol (a minimum of 18-24 hours prior to enrollment)…” We have clarified the labeling of study subjects, with the following addition to the Methods section (lines 104-107) “These patients were from two larger cohorts named “SEGD” and “PIC”, both of which had paired biopsy and blood specimens. However, histology was performed from “PIC” patients only and flow cytometry was done in “SEGD” patients only (Supplementary table 1).”

We have also clarified on the Ogawa and Inaba mentioned in Suppl Table 1, in both Methods (line 149-150), “To examine the vibriocidal antibody response to the two serotypes of V. cholerae O1 (Ogawa and Inaba), we performed the vibriocidal assay as previously described”, and in Table legend, “Demographics and vibriocidal antibody titers (to the two V. cholerae O1 serotypes) of study subjects.”

15. The labels on the gating strategy and % on histograms in fig 2E are impossible to read.

Response: This has now been modified for clarity.

16. Data on peripheral MAIT cells (which the authors state did not correlate with Stool markers) should be shown in Fig 3.

Response: Data on peripheral MAIT cells has now been added as Fig 3C-D and the figure legend is modified (line 418) as “(C-D) shows the correlation of PBMC MAITs with MPO (C) and with AAT (D).”

17. Why was the B7 integrin expression only examined at day 7 (Fig 4), and why only on 4 patients? This figure should also show the expression for healthy controls. By itself this data is not meaningful to assess the gut homing capacity of MAIT cells in cholera and should be expanded or removed.

Response: Thank you for this critique. Given the lack of integrin β7 data from all participants, lack of healthy controls, and the limited information gained, we removed these data from our manuscript.

18.Labels on Fig 5 are hard to read, particularly the different samples.

Response: Labels on the previous Fig 5 (now Fig 4) have now been modified for clarity.

19. Line 241 should state that the dominant clone was found in one of the two patients examined. The text also states that 46 clones were found in PBMC and LPL, but this data is not shown. Fig 1A should state (or in a table) how many times each sequence was identified. Similarly for convalescent clone data (line 248).

Response: We have now included Supplemental Table 3 demonstrating all paired TCRαβ sequences identified in each patient, and text added to Results. Of note, the majority of cells sequenced had paired TCR sequences, with the exception of cells from the day 2 LPL sample, which only had one paired sequence, and thus the majority of TCR sequenced was unpaired and are plotted in Figures 4A, 4B, and 4C. We have now changed in Lines 250-255, “Unfortunately, despite many unpaired TCRa and TCRb sequences available from the acute day 2 LPL sample (Figure 4A-C), there was only one paired TCRab clone sequenced (Supplemental Table 3), and thus comparisons between LPL and PBMC clonality could not be made based on paired TCR reads.”

20. In fig 5B, the majority of clones from patient 1 express TRAJ34. This seems unusual for MAIT cells, although has been described in an “atypical”, TRAV1-2- MAIT cells (Gherardin et al. Immunity 2016, Koay et al. N. Comm 2019) and in one clone expanded from HIV infected individuals after stimulation with E. coli (Trivedi et al., ImunoHorizons 2021). Can the authors comment on why this may be and whether this is likely to be a generalisable phenomenon?

Response: Thank you for making this observation. As you noted, we have observed TRAJ34 pairing with TRAV1-2 in both this study and in our study of MAIT cells in people living with HIV. Further work is needed to examine the significance of this observation, including its generalizability. We have added to the Discussion (line 344), “thus further studies would be needed to confirm our observations regarding the TCR usage between LPL and blood MAITs, including the significance and generalizability of the TRAJ34 clones isolated.”

21. The data in Fig 5E doesn’t make sense. Frequencies should add up to 100 (line 254-259 in text).

Response: We apologize for this confusing figure. The Y axis is the absolute number of times (not percentage) of CDR3b with a particular amino acid length. This has been corrected in results (line 263 is updated to number instead of frequency), the y-axis title, and the figure legend (line 429) has been modified to, “x-axis shows length distribution of amino acids and y axis shows number of times CDR3β sequence was found with that amino acid length.”

22. Line 275: [35] is the wrong reference.

Response: Thank you. This reference has been corrected to ref 4.

**Reviewer #3:** In this study, Bhuiyan et al investigate MAIT cell frequencies, phenotype and TCR rearrangements during acute and convalescent cholera infection. The authors should be commended for using intestinal material and paired data from the same individuals, as these tell much more than only circulating cells or animal models. However, it also creates problems such as limited tissue availability restricting the analyses that can be performed. It may also be hard to convince acutely ill patients to perform endoscopy for research purposes. This is probably to cause of one of the major limitations of the study, the low number of individuals included in the different analyses. Another major weakness is the unclear identification of MAIT cells in all the flow cytometry analyses.

Major points:

1. The use of Va7.2 and CD161 to identify mucosal MAIT cells is somewhat problematic, as there are other populations of CD161-expressing T cells in the intestinal mucosa. The authors would need to confirm their findings using MR1 tetramers and CD161 to identify MAIT cells. The MR1 tetramers are now available from the NIH tetramer facility since several years.

Furthermore, MAIT cells are said to be defined as CD3+CD4- cells expressing the MAIT TCR and CD161 (line 150). However, in the representative staining shown in Fig. 2, all T cells are included when the MAIT gate is set. Likewise, the manuscript consistently gives the number of MAIT cells per CD3+ cells, not CD8+ or CD3+CD4-, and this creates some confusion as to which gates were actually used. As Th17 cells and Treg can express CD161, some of them (expressing the Va7.2 as part of another non-MAIT TCR) may also have been included in the analyses.

Response: Unfortunately, during the period of this study (2012-2014), the MR1-tetramer was not available. We have added to the Discussion (lines 348-353), “our identification of MAIT cells was based on Va7.2 antibody, as the MR1-tetramer was not available at the time that this study was conducted (2012-2014). Thus, our results are subject to lack of sensitivity to detect activated MAIT cells, given their potential for down-regulation of CD161 and TCR alpha chain [51], and may also include a small portion of non-MR1-restricted T cells.”

We have examined the flow cytometry data with and without CD4 involved in the gating strategy and this did not change any of our findings (no differences between day 2 and day 30). To address comments above and also posed by other reviewers, to be more consistent with MAIT identification by immunohistochemistry, we have revised our identification of MAIT cells by flow to not include CD4 gating (i.e. MAITs are CD3^+^CD161^hi^Vα7.2^+^ cells), and we have made this changed in Methods section (line 129), and revised our Results section and figures to consistently give frequency of MAIT cells as % of CD3+ cells.

2. The IF staining in Fig. 1A is hard to interpret. Please provide a close up of the indicated MAIT cells, to show how they can be distinguished from the background. Please also show the DAPI stain so that individual cells can be identified.

Response: Thank you for your suggestion. Unfortunately, this is the highest resolution and magnification we were able to obtain. These images were obtained and saved with this resolution in 2013, and we are unable to improve the resolution of the saved images.

3. CD69 is a good activation marker for circulating T cells. In tissues however, CD69 also marks tissue-resident memory T cells, and is thus less suitable. Therefore, the authors may need to modify their statements on activation status in intestinal MAIT cells.

Please show the FMO controls for CD38 and CD69 in the intestinal samples in Fig. 2E.

Response: Thank you for these suggestions. We agree that CD69 is a marker of tissue-resident memory T cells, and thus we have focused the statements on activation status on our findings with CD38+ MAIT cells. We have modified Results (line 216-217) to, “We found no significant differences in CD38^+^ MAIT cells and CD69^+^ MAIT cells between duodenal MAITs at day 2 and day 30,” and Lines 314 and 365 now modified to “CD38^+^ MAIT cells”. We have now shown FMO controls in Fig 2E.

4. There are clear differences with regard to changes in MAIT cell frequencies during the course of disease between IHC and flow cytometry. This should be discussed.

Furthermore, the authors’ previous finding of highly activated circulating MAIT cells on day 7 (ref 14) was not confirmed in the present study. This might also deserve some comments in the discussion.

The authors conclude that “MAIT cells are highly activated and present in the lamina propria of the duodenum during cholera”. However, the same can be said about the convalescent stage, as there is actually no difference between day 2 and day 30. A more correct statement would be that lamina propria MAIT cells are more activated than those in the blood.

There are several studies on MAIT cell activity following Salmonella infection or vaccination (for example Howson et al, Nature Comm 2018 and Salerno-Goncalves et al Front Immunol 2017) that could also be included in the discussion.

Response:

Regarding the discrepancy between flow cytometry and immunohistochemistry, we have now added to the discussion (line 291-298) as follows “A potential explanation for this discrepancy include differences in sample preparation for flow cytometry and immunohistochemistry, as embedding and fixation of some antigenic epitopes could be lost during the processing and preparation of specimens, and collagenase treatment used to prepare samples for flow cytometry can also impact expression of surface markers [36, 37]. Furthermore, there is a lower detection threshold of flow cytometry compared with immunohistochemistry, whereby dim antigen expression may be easily distinguished from background. Such discrepancies between flow cytometric and immunohistochemical results have been reported by others [38-40].”

Regarding the discrepancy in day 7 activation of PBMC MAIT cells between this and the previous study, as our primary objective was to compare duodenal LPL with circulating PBMC, we did not make this comparison initially. In response to your query, we have since performed a statistical analysis by Wilcoxon signed ranked test between day 2 and day 7 CD38+ in PBMC, and found a statistically non-significant (p = 0.07) difference, which may be due to the smaller sample size of this current study.

We have changed our Discussion (Line 315-316) to, “Taken together, MAIT cells are present in the lamina propria of the duodenum during cholera, and more activated than those in the blood.” We have also changed the Abstract (Line 46) accordingly.

Thank you for the suggestion to include Salmonella MAIT papers. We have now included a discussion on MAIT cells following Salmonella infection. Line 309-313 reads “In addition to this, a decrease in circulating MAIT cell frequencies has been reported previously in studies of live S. Typhi [46, 47] and live-attenuated Shigella dysenteriae 1 vaccine[7], suggesting that circulating MAIT cells may decrease in frequency in the blood as they move to locally inflamed and infected tissues.“ We have also included in discussion (line 334-335), ”It has been shown that MAIT cells undergo clonal expansion after Salmonella enterica serovar Paratyphi A infection [47].”

5. In Fig. 3, why not include the MAIT cell frequencies obtained from the IHC data? These are the patients that actually did show a change in MAIT cell frequencies during infection.

Response: Unfortunately, the MPO and AAT assays were only run on the cohort of patients that we performed flow cytometry of LPL on, not the cohort that we performed IHC on. We have clarified in the Methods section (lines 155-156), “In a subset of patients from the flow cytometry cohort whom we had data from both day 2 and day 30 “, and results section (Lines 227-229), “…in six of the eight cholera patients from whom we had paired days 2 and 30 LPL MAIT cell data from the flow cytometry cohort, and seven of the eight from whom we had paired PBMC data.”

6. To evaluate the data presented in Fig. 4 properly, it would be better to show also the results from day 2 and day 30.

Response: Given the lack of integrin β7 data from controls, and the limited information gained, we have decided to remove these data from our manuscript.

We hope we have been able to satisfactorily respond to the comments raised by the reviewers and that the paper is now suitable for acceptance in PLOS Neglected Tropical Diseases.

Thank you again for your consideration of this manuscript.

Sincerely yours,

Daniel T. Leung, MD: Corresponding author

Associate Professor

Division of Infectious Diseases

University of Utah School of Medicine

30 N 1900 E, RM 4C416

Salt Lake City, UT, USA

Email: [Daniel.Leung@utah.edu](mailto:Daniel.Leung@utah.edu)
