# Supplementary material for: Mucosal-Associated Invariant T (MAIT) cells are highly activated in duodenal tissue of humans with Vibrio cholerae O1 infection: A preliminary report
Source: PLoS Negl Trop Dis. 2022 May 12;16(5):e0010411. doi: 10.1371/journal.pntd.0010411 (PMC9129025; doi:10.1371/journal.pntd.0010411)
Supplement: Peer review history

# Peer review: Decision Letter 0

**DOI:** 10.1371/journal.pntd.0010411.r001

**Authors:** Alfredo G Torres, Khitam Muhsen

16 Jul 2021

Dear Dr. Leung,

Thank you very much for submitting your manuscript "Mucosal-Associated Invariant T (MAIT) Cells are Highly Activated in Duodenal Tissue of Humans with Vibrio cholerae O1 Infection" for consideration at PLOS Neglected Tropical Diseases. As with all papers reviewed by the journal, your manuscript was reviewed by members of the editorial board and by several independent reviewers. In light of the reviews (below this email), we would like to invite the resubmission of a significantly-revised version that takes into account the reviewers' comments. 

We cannot make any decision about publication until we have seen the revised manuscript and your response to the reviewers' comments. Your revised manuscript is also likely to be sent to reviewers for further evaluation.

When you are ready to resubmit, please upload the following:

[1] A letter containing a detailed list of your responses to the review comments and a description of the changes you have made in the manuscript. Please note while forming your response, if your article is accepted, you may have the opportunity to make the peer review history publicly available. The record will include editor decision letters (with reviews) and your responses to reviewer comments. If eligible, we will contact you to opt in or out.

[2] Two versions of the revised manuscript: one with either highlights or tracked changes denoting where the text has been changed; the other a clean version (uploaded as the manuscript file). 

Important additional instructions are given below your reviewer comments.

Please prepare and submit your revised manuscript within 60 days. If you anticipate any delay, please let us know the expected resubmission date by replying to this email. Please note that revised manuscripts received after the 60-day due date may require evaluation and peer review similar to newly submitted manuscripts.

Thank you again for your submission. We hope that our editorial process has been constructive so far, and we welcome your feedback at any time. Please don't hesitate to contact us if you have any questions or comments.

Sincerely,

Khitam Muhsen

Guest Editor

PLOS Neglected Tropical Diseases

Alfredo Torres

Deputy Editor

PLOS Neglected Tropical Diseases

***********************

Reviewer's Responses to Questions

**Key Review Criteria Required for Acceptance?**

As you describe the new analyses required for acceptance, please consider the following:

**Methods**

-Are the objectives of the study clearly articulated with a clear testable hypothesis stated?

-Is the study design appropriate to address the stated objectives?

-Is the population clearly described and appropriate for the hypothesis being tested?

-Is the sample size sufficient to ensure adequate power to address the hypothesis being tested?

-Were correct statistical analysis used to support conclusions?

-Are there concerns about ethical or regulatory requirements being met?

Reviewer #1: Please see "Summary and General Comments" section

Reviewer #2: The population is not fully described, healthy controls are lacking and the sample sizes are small in some analyses. See full comments below.

Reviewer #3: The objectives are reasonably outlined, and the study design appropriate.

The population is well described and also appropriate for the study. The main worry here is the population size, as the inter-individual variation is large. The number of study patients is low, which makes it hard to make solid conclusions.

It is unclear from the text which statistical methods have actually been used in Fig. 1 and Fig. 4, the M&M section states Wilcoxon and the figure legends t-test.

No ethical concerns.

--------------------

**Results**

-Does the analysis presented match the analysis plan?

-Are the results clearly and completely presented?

-Are the figures (Tables, Images) of sufficient quality for clarity?

Reviewer #1: Please see "Summary and General Comments" section

Reviewer #2: There are unexplained inconsistencies between the data presented in different figures.

See full comments below.

Reviewer #3: The analysis is according to plan. 

There are some issues with regard to result presentation. The IF staining in Fig. 1A is hard to interpret. Please provide a close up of the indicated MAIT cells, to show how they can be distinguished from the background. Please also show the DAPI stain so that individual cells can be identified. 

There is also a lack of FMO controls in Fig. 2, which would be needed to judge the cut off for positive CD38 and CD39 staining.

Furthermore, the labeling of the axes in the dot plots in Fig. 2 and of the samples in Fig. 5A-D is very hard to read and clarity can be improved.

--------------------

**Conclusions**

-Are the conclusions supported by the data presented?

-Are the limitations of analysis clearly described?

-Do the authors discuss how these data can be helpful to advance our understanding of the topic under study?

-Is public health relevance addressed?

Reviewer #1: Please see "Summary and General Comments" section

Reviewer #2: Conclusions are not always fully supported. See full comments below.

Reviewer #3: The low patient number makes it hard to draw solid conclusions. This limitation in mentioned, but not elaborated. The conflicting results from IHC and flow cytometry and how data can be used to advance understanding of the topic are not really discussed either.

--------------------

**Editorial and Data Presentation Modifications?**

Use this section for editorial suggestions as well as relatively minor modifications of existing data that would enhance clarity. If the only modifications needed are minor and/or editorial, you may wish to recommend “Minor Revision” or “Accept”. 

Reviewer #1: (No Response)

Reviewer #2: Several small modifications. See fully comments (under minor comments heading) below.

Reviewer #3: 1. The first three references are quite old, and there are more recent reviews on the topic that could be used to introduce the reader to the topic of MAIT cells.

2. The first paragraph of the result section would be better suited in the materials and methods. Please also define VE blood group antigens in Suppl. Table 1.

3. The M&M sections states that Wilcoxon was used to compare values before and after infection, but in the legends to Fig. 1 and 4 it say paired t-test. Please clarify.

4. In Fig. 1C, only 1-4% of lamina propria lymphocytes are CD3+. This is surprisingly low, has there maybe been a mistake in the labeling of the y-axis?

5. In the text (line 193 and 216) it says that 7 patients were available for biopsies day 30, but in the corresponding plot in Fig. 2A, 8 individuals are shown. Please clarify.

6. In M&M, it is stated that cells were sorted from one donor for TCR analysis (line 146) while in the result section, data from two individuals are presented (line 237). Please clarify. The second patient appears to be biopsied at day 180, which is not mentioned in the M&M. Furthermore, how were these patients selected?

7. Reference 32 and 33 are the same.

--------------------

**Summary and General Comments**

Use this section to provide overall comments, discuss strengths/weaknesses of the study, novelty, significance, general execution and scholarship. You may also include additional comments for the author, including concerns about dual publication, research ethics, or publication ethics. If requesting major revision, please articulate the new experiments that are needed.

Reviewer #1: The study by Bhuiyan et al. characterized MAIT cells present in duodenal biopsies and their paired peripheral blood samples in patients with culture-confirmed Vibrio cholerae O1 infection. The manuscript is sound, interesting, and presents novel findings reported straightforwardly.

A few suggestions are below:

1. Please transfer the paragraph containing the demographics from the “Results” (lines 171-177) to the “methods” section (i.e., “Study population and sample collection” section) of the manuscript. 

2. Please explain, in the manuscript, the rationale for identifying MAIT cells using different markers (e.g., CD161 for blood and IL-18Rα for duodenal biopsies). The readers may be confused by a choice that is not explained.

3. Figure 1. Panel A would benefit from a figure with higher resolution and magnification. 

4. Please confirm that day 2 and 30 biopsies were collected from the same area of the duodenum and not randomly. Add a statement clarifying this issue into the manuscript. Also, discuss further the discrepancy between duodenum MAIT cell frequencies obtained by immunochemistry (Fig. 1B) and those obtained by flow cytometry (Fig. 2A). Is it a sampling issue? Or experiment artifact? Collagenase might have impacted the expression of MAIT specific markers... Further immunochemistry stainings are necessary to clarify this issue.

5. References 38 and 39 are the same.

6. The discussion lacks publications showing MAIT cell kinetics (e.g., PMID: 23898209, PMID: 32811991, PMID: 28428786). Given that the authors dwell on the effects of MAIT cells in the duodenal biopsies and blood, it would perhaps be helpful if, at the end of the discussion, they committed to a statement on the pattern of the MAIT cell kinetics and its implications on cholera pathology.

7. Since no kinetic changes occurred in either blood or duodenum, please discuss the need for extra time points to establish the impact of MAIT cells on cholera infection

8. Material & Methods. There is no statement about blood collection at days 7 and 180, biopsies at day 180, or integrin β7 staining. If an oversight, please fix it.

9. Fix typo. “Results” section states, “The majority of MAIT cells were identified in the lamina propria, with no cells identified in the villous and crypt epithelia (lines 183 & 184), but the discussion describes, “we demonstrated that the vast majority of MAIT cells are in the lamina propria, predominantly in the crypts, and rarely in the epithelia.”( lines 271-273). In the discussion, do you imply beneath the crypts?

Reviewer #2: Summary

MAIT cells are known to be activated by diverse microbes and several mouse studies have shown their role in protecting against bacterial infection, but also in driving pathology in chronic infection. Studies of MAIT cells in humans during infection, particularly at mucosal sites, are currently limited.

Here, Bhuiyan and colleagues assess MAIT cell numbers in acute and convalescent Vibrio cholerae infection in duodenal biopsies and bloods from a small cohort of patients. The topic is interesting and the study would potentially add important information to the field. However, much of the data presented seems preliminary (for example the clonal expansion observed in one patient). He study lacks healthy control samples (which could easily be included for PBMCs if not biopsies). There is a relatively small number of patient used, and only small subsets of these analysed for the data shown in different figures (5 patients for some).

There are also inconsistencies between data from flow cytometry and histology that are not explained, and these then also affect downstream analyses. 

Detailed comments below offer suggestions to improve the study.

Major comments

1. The histology images shown in Fig 1A is of low resolution (even in downloaded .tif file). Some of the arrows for Va7.2 staining do not appear to be pointing to cells. It is hard to assess from the histology presented if statements about location in the LP are correct (line 183).

2. How was the quantitation done in Fig 1B and C? i.e. how many sections, from how many biopsies were enumerated for each patient? Was the operator blinded? What is meant by “paired duodenal biopsies” in line 181?

3. How do the authors explain the inconsistency in terms of %MAITs of CD3+ T cells in duodenal samples between data shown in Fig 1B vs Fig 2A and Fig 1C vs 2B? In Fig. 1B, authors claimed that frequency of MAIT cells was significantly higher at D2 compare to D30. However, no difference was observed in Fig 2A.

4. It is a worry that the authors claim to show MAIT cell frequencies increase in acute infection vs convalescence (line 282) based on histology data, but that they “were unable to replicate this finding using flow cytometric analysis…”. This is cherry-picking since they could equally state there was no difference based on the flow data. Given that neither assessment used MR1 tetramers, which would precisely detect MAIT cells, can the authors be sure that the markers used do not change expression during activation leading to loss of signal and apparent decrease in MAIT cells?

5. In flow cytometry data, MAIT cells are defined as CD3+CD4−CD161hiVα7.2+ cells. However, some MAIT cells express CD4. It would be interesting to look at CD4 (and CD8) coreceptor expression if this data is available.

6. What is the percentage of MAIT cells among CD3+ cell in cholera-free duodenal tissue? Without such data, it is hard to claim that “MAIT cell frequencies are significantly increased during acute infection compared to convalescence”.

7. For Fig. 2C and D, duodenal MAIT cells highly express CD38 and CD69 at both acute and convalescent stages of cholera. Is this an intrinsic property of duodenal (or tissue) MAIT cells or induced by bacterial infection? In particular, CD69 expression is known to be high in tissues compared to PBMCs. In order to claim that duodenal MAIT cells in cholera infection are activated based on these markers (line 137), it is necessary to examine CD38 and CD69 expression on MAIT cells from uninfected duodenal samples.

8. For Fig. 2E, isotype or FMO control should be shown for CD69 and CD38 staining

9. For Fig. 4, FACS plots showing β7 expression on peripheral MAIT and non-MAIT cells should be included. Β7 data from healthy control is also required.

10. For Fig. 5A, is the dominant MAIT cell clone in the LPL a result of selective proliferation? Or preferential tissue homing? Again, uninfected controls would be welcomed here.

Minor comments

11. Line 60. “The ligand for MAIT cells…” This should be “antigen” and there is more than one described.

12. TNFa should be TNF consistent with current naming convention.

13. The methods are light on detail and insufficient for others to repeat. For example, antibodies should be listed with clone name, catalogue numbers and concentrations used for staining.

14. The patient information should be more detailed. For example, Inaba and Ogawa are only mentioned in Supp Table 1 and not explained. Are these patients part of a larger study? What are SEGD and PIC in the patient numbers? Which patient samples were used for FACS vs histology? The antibiotics given should be described along with the timing relative to biopsies (line 94). Is the use of these antibiotics known or anticipated to affect the MAIT cell response?

15. The labels on the gating strategy and % on histograms in fig 2E are impossible to read.

16. Data on peripheral MAIT cells (which the authors state did not correlate with Stool markers) should be shown in Fig 3.

17. Why was the B7 integrin expression only examined at day 7 (Fig 4), and why only on 4 patients? This figure should also show the expression for healthy controls. By itself this data is not meaningful to assess the gut homing capacity of MAIT cells in cholera and should be expanded or removed.

18.Labels on Fig 5 are hard to read, particularly the different samples. 

19. Line 241 should state that the dominant clone was found in one of the two patients examined. The text also states that 46 clones were found in PBMC and LPL, but this data is not shown. Fig 1A should state (or in a table) how many times each sequence was identified. Similarly for convalescent clone data (line 248). 

20. In fig 5B, the majority of clones from patient 1 express TRAJ34. This seems unusual for MAIT cells, although has been described in an “atypical”, TRAV1-2- MAIT cells (Gherardin et al. Immunity 2016, Koay et al. N. Comm 2019) and in one clone expanded from HIV infected individuals after stimulation with E. coli (Trivedi et al., ImunoHorizons 2021). Can the authors comment on why this may be and whether this is likely to be a generalisable phenomenon? 

21. The data in Fig 5E doesn’t make sense. Frequencies should add up to 100 (line 254-259 in text).

22. Line 275: [35] is the wrong reference.

Reviewer #3: In this study, Bhuiyan et al investigate MAIT cell frequencies, phenotype and TCR rearrangements during acute and convalescent cholera infection. The authors should be commended for using intestinal material and paired data from the same individuals, as these tell much more than only circulating cells or animal models. However, it also creates problems such as limited tissue availability restricting the analyses that can be performed. It may also be hard to convince acutely ill patients to perform endoscopy for research purposes. This is probably to cause of one of the major limitations of the study, the low number of individuals included in the different analyses. Another major weakness is the unclear identification of MAIT cells in all the flow cytometry analyses. 

Major points:

1. The use of Va7.2 and CD161 to identify mucosal MAIT cells is somewhat problematic, as there are other populations of CD161-expressing T cells in the intestinal mucosa. The authors would need to confirm their findings using MR1 tetramers and CD161 to identify MAIT cells. The MR1 tetramers are now available from the NIH tetramer facility since several years.

Furthermore, MAIT cells are said to be defined as CD3+CD4- cells expressing the MAIT TCR and CD161 (line 150). However, in the representative staining shown in Fig. 2, all T cells are included when the MAIT gate is set. Likewise, the manuscript consistently gives the number of MAIT cells per CD3+ cells, not CD8+ or CD3+CD4-, and this creates some confusion as to which gates were actually used. As Th17 cells and Treg can express CD161, some of them (expressing the Va7.2 as part of another non-MAIT TCR) may also have been included in the analyses.

2. The IF staining in Fig. 1A is hard to interpret. Please provide a close up of the indicated MAIT cells, to show how they can be distinguished from the background. Please also show the DAPI stain so that individual cells can be identified. 

3. CD69 is a good activation marker for circulating T cells. In tissues however, CD69 also marks tissue-resident memory T cells, and is thus less suitable. Therefore, the authors may need to modify their statements on activation status in intestinal MAIT cells. 

Please show the FMO controls for CD38 and CD69 in the intestinal samples in Fig. 2E.

4. There are clear differences with regard to changes in MAIT cell frequencies during the course of disease between IHC and flow cytometry. This should be discussed. 

Furthermore, the authors’ previous finding of highly activated circulating MAIT cells on day 7 (ref 14) was not confirmed in the present study. This might also deserve some comments in the discussion.

The authors conclude that “MAIT cells are highly activated and present in the lamina propria of the duodenum during cholera”. However, the same can be said about the convalescent stage, as there is actually no difference between day 2 and day 30. A more correct statement would be that lamina propria MAIT cells are more activated than those in the blood.

There are several studies on MAIT cell activity following Salmonella infection or vaccination (for example Howson et al, Nature Comm 2018 and Salerno-Goncalves et al Front Immunol 2017) that could also be included in the discussion.

5. In Fig. 3, why not include the MAIT cell frequencies obtained from the IHC data? These are the patients that actually did show a change in MAIT cell frequencies during infection.

6. To evaluate the data presented in Fig. 4 properly, it would be better to show also the results from day 2 and day 30.

--------------------

PLOS authors have the option to publish the peer review history of their article (what does this mean?). If published, this will include your full peer review and any attached files.

If you choose “no”, your identity will remain anonymous but your review may still be made public.

**Do you want your identity to be public for this peer review?** For information about this choice, including consent withdrawal, please see our Privacy Policy.

Reviewer #1: No

Reviewer #2: No

Reviewer #3: Yes: Marianne Quiding Järbrink

Figure Files:

While revising your submission, please upload your figure files to the Preflight Analysis and Conversion Engine (PACE) digital diagnostic tool, https://pacev2.apexcovantage.com. PACE helps ensure that figures meet PLOS requirements. To use PACE, you must first register as a user. Then, login and navigate to the UPLOAD tab, where you will find detailed instructions on how to use the tool. If you encounter any issues or have any questions when using PACE, please email us at figures@plos.org.

Data Requirements:

Please note that, as a condition of publication, PLOS' data policy requires that you make available all data used to draw the conclusions outlined in your manuscript. Data must be deposited in an appropriate repository, included within the body of the manuscript, or uploaded as supporting information. This includes all numerical values that were used to generate graphs, histograms etc.. For an example see here: http://www.plosbiology.org/article/info%3Adoi%2F10.1371%2Fjournal.pbio.1001908#s5.

Reproducibility:

To enhance the reproducibility of your results, we recommend that you deposit your laboratory protocols in protocols.io, where a protocol can be assigned its own identifier (DOI) such that it can be cited independently in the future. Additionally, PLOS ONE offers an option to publish peer-reviewed clinical study protocols. Read more information on sharing protocols at https://plos.org/protocols?utm_medium=editorial-email&utm_source=authorletters&utm_campaign=protocols

# Author comment: Author response to Decision Letter 0

**DOI:** 10.1371/journal.pntd.0010411.r002

22 Sep 2021

Attachment Submitted filename: pNTD 21-00600 Response to reviewers.docx Click here for additional data file.

# Peer review: Decision Letter 1

**DOI:** 10.1371/journal.pntd.0010411.r003

**Authors:** Alfredo G Torres, Khitam Muhsen

12 Oct 2021

Dear Dr. Leung,

Thank you very much for submitting your manuscript "Mucosal-Associated Invariant T (MAIT) Cells are Highly Activated in Duodenal Tissue of Humans with Vibrio cholerae O1 Infection" for consideration at PLOS Neglected Tropical Diseases. As with all papers reviewed by the journal, your manuscript was reviewed by members of the editorial board and by several independent reviewers. In light of the reviews (below this email), we would like to invite the resubmission of a significantly-revised version that takes into account the reviewers' comments. 

Given the sample size in this study, the preliminary nature of the data it is recommended to add "a preliminary report" to the title. In addition, more details on the sample size, age and sex of the patients should be added to the abstract and summary. 

In the conclusions (both abstract / discussion) please highlight the small sample size. Please also moderate the conclusions, (especially when some results were not statistically significant at p<0.05) and highlight the preliminary nature of the report. 

Figures legend – please enter the number of samples tested in each group/time points. Figure 2: When using ANOVA please check if the underling assumptions of ANOVA were fulfilled, otherwise please use non-parametric test.

We cannot make any decision about publication until we have seen the revised manuscript and your response to the reviewers' comments. Your revised manuscript is also likely to be sent to reviewers for further evaluation.

When you are ready to resubmit, please upload the following:

[1] A letter containing a detailed list of your responses to the review comments and a description of the changes you have made in the manuscript. Please note while forming your response, if your article is accepted, you may have the opportunity to make the peer review history publicly available. The record will include editor decision letters (with reviews) and your responses to reviewer comments. If eligible, we will contact you to opt in or out.

[2] Two versions of the revised manuscript: one with either highlights or tracked changes denoting where the text has been changed; the other a clean version (uploaded as the manuscript file). 

Important additional instructions are given below your reviewer comments.

Please prepare and submit your revised manuscript within 60 days. If you anticipate any delay, please let us know the expected resubmission date by replying to this email. Please note that revised manuscripts received after the 60-day due date may require evaluation and peer review similar to newly submitted manuscripts.

Thank you again for your submission. We hope that our editorial process has been constructive so far, and we welcome your feedback at any time. Please don't hesitate to contact us if you have any questions or comments.

Sincerely,

Khitam Muhsen

Guest Editor

PLOS Neglected Tropical Diseases

Alfredo Torres

Deputy Editor

PLOS Neglected Tropical Diseases

***********************

Given the sample size in this study, the preliminary nature of the data it is recommended to add "a preliminary report" to the title. In addition, more details on the sample size, age and sex of the patients should be added to the abstract and summary. 

In the conclusions (both abstract / discussion) please highlight the small sample size. Please also moderate the conclusions, (especially when some results were not statistically significant at p<0.05) and highlight the preliminary nature of the report. 

Figures legend – please enter the number of samples tested in each group/time points. Figure 2: When using ANOVA please check if the underling assumptions of ANOVA were fulfilled, otherwise please use non-parametric test.

Reviewer's Responses to Questions

**Key Review Criteria Required for Acceptance?**

As you describe the new analyses required for acceptance, please consider the following:

**Methods**

-Are the objectives of the study clearly articulated with a clear testable hypothesis stated?

-Is the study design appropriate to address the stated objectives?

-Is the population clearly described and appropriate for the hypothesis being tested?

-Is the sample size sufficient to ensure adequate power to address the hypothesis being tested?

-Were correct statistical analysis used to support conclusions?

-Are there concerns about ethical or regulatory requirements being met?

Reviewer #1: (No Response)

Reviewer #2: small sample size, but noting these samples are difficult to obtain and this is highlighted in the manuscript.

Reviewer #3: The weaknesses of the study have now been better spelled out in the manuscript.

--------------------

**Results**

-Does the analysis presented match the analysis plan?

-Are the results clearly and completely presented?

-Are the figures (Tables, Images) of sufficient quality for clarity?

Reviewer #1: (No Response)

Reviewer #2: (No Response)

Reviewer #3: The inconsistencies in the result section have been clarified in the revised version of the manuscript. However, immunofluorescence pictures are not of an acceptable quality.

--------------------

**Conclusions**

-Are the conclusions supported by the data presented?

-Are the limitations of analysis clearly described?

-Do the authors discuss how these data can be helpful to advance our understanding of the topic under study?

-Is public health relevance addressed?

Reviewer #1: (No Response)

Reviewer #2: see comments below

Reviewer #3: These issues are adequately addressed in the revised manuscript.

--------------------

**Editorial and Data Presentation Modifications?**

Use this section for editorial suggestions as well as relatively minor modifications of existing data that would enhance clarity. If the only modifications needed are minor and/or editorial, you may wish to recommend “Minor Revision” or “Accept”. 

Reviewer #1: (No Response)

Reviewer #2: see comments below

Reviewer #3: (No Response)

--------------------

**Summary and General Comments**

Use this section to provide overall comments, discuss strengths/weaknesses of the study, novelty, significance, general execution and scholarship. You may also include additional comments for the author, including concerns about dual publication, research ethics, or publication ethics. If requesting major revision, please articulate the new experiments that are needed.

Reviewer #1: All the points raised by this reviewer have been addressed satisfactorily.

Reviewer #2: The authors have revised their manuscript addressing most comments resulting in an improved manuscript. They have removed some less convincing data (previous figure 4). Some issues remain as listed below.

Previous comment. 1. The histology images shown in Fig 1A is of low resolution (even in downloaded.tif file). Some of the arrows for Va7.2 staining do not appear to be pointing to cells. It is hard to assess from the histology presented if statements about location in the LP are correct (line 183). 

Response: Thank you for your suggestion. Unfortunately, this is the highest resolution and magnification we were able to obtain. These images were obtained and saved with this resolution in 2013, and we are unable to improve the resolution of the saved images. 

Remaining issues: the response does not address the main issues. It is still difficult to determine the validity of data presented in B and C when some arrows do not appear to be pointing to cells. Crypt and villi structures are hard to see. Noting the difficulty in sourcing these samples the authors could at least list this as a limitation and ensure their conclusions based on these results are appropriately phrased.

Previous comment. 2. How was the quantitation done in Fig 1B and C? i.e. how many sections, from how many biopsies were enumerated for each patient? Was the operator blinded? What is meant by “paired duodenal biopsies” in line 181? 

Response: We have added to the Methods section (lines 145-147), “For each patient, a single operator, using ImageJ software, enumerated both MAIT and CD3+ cells from a single biopsy section at each time point from paired (available at both day 2 and day 30) samples.” 

Remaining issues: The response does not fully address the previous comment. Was the operator blinded to the time points for each biopsy?

What were the absolute numbers counted? Were more biopsies available? What was the variation in numbers between patients? Drawing conclusions from such small numbers of biopsies is problematic in this reviewer’s opinion, particularly if the operator was not blinded to the grouping.

Previous comment. 3. How do the authors explain the inconsistency in terms of %MAITs of CD3+ T cells in duodenal samples between data shown in Fig 1B vs Fig 2A and Fig 1C vs 2B? In Fig. 1B, authors claimed that frequency of MAIT cells was significantly higher at D2 compare to D30. However, no difference was observed in Fig 2A. 

Response: To address the discrepancy between flow cytometry and immunohistochemistry, we have now added to the discussion (line 291-298) as follows, “A potential explanation for this discrepancy include differences in sample preparation for flow cytometry and immunohistochemistry, as embedding and fixation of some antigenic epitopes could be lost during the processing and preparation of specimens, and collagenase treatment used to prepare samples for flow cytometry can also impact expression of surface markers [36, 37]. Furthermore, there is a lower detection threshold of flow cytometry compared with immunohistochemistry, whereby dim antigen expression may be easily distinguished from background. Such discrepancies between flow cytometric and immunohistochemical results have been reported by others [38-40].” 

Remaining issues: It is unclear how these factors would differentially affect the results at day 2 and day 30.

Additionally, the histology differences are no longer reported as significant (p = 0.06) as the authors have changed the statistical test used, and thus claims of differences between the two time points should be avoided.

Previous comment. 4. It is a worry that the authors claim to show MAIT cell frequencies increase in acute infection vs convalescence (line 282) based on histology data, but that they “were unable to replicate this finding using flow cytometric analysis...”. This is cherry-picking since they could equally state there was no difference based on the flow data. Given that neither assessment used MR1 tetramers, which would precisely detect MAIT cells, can the authors be sure that the markers used do not change expression during activation leading to loss of signal and apparent decrease in MAIT cells? 

Response: Unfortunately, during the period of this study (2012-2014), the MR1-tetramer was not available. Thus, we initially identified MAIT as CD3+CD4−CD161hiVα7.2+ for flow cytometry as we have used in our earlier publication (Ref 14). For immunohistochemistry, we used the staining markers for MAIT cells published previously by other groups (PMID 21084709, 24450998). 

We have now added this statement to the Methods section (line 136-138): “In line with immunohistochemical methods from reports available at time of the experiments [16, 17],” To be more consistent with immunohistochemistry methods, we have revised our identification of MAIT cells by flow to not include CD4 gating (i.e. MAITs are CD3+CD161hiVα7.2+ cells), and we have made this changed in Methods section (line 129). We have now highlighted the discrepancy between flow cytometry and immunohistochemistry findings in the Discussion, as quoted in response to the question above. 

Remaining issues: The flow cytometry data does not appear to be changed (Fig 2) to reflect the change in gating. As above, claims of “differences” should be avoided when not significant.

Previous comment. 5. In flow cytometry data, MAIT cells are defined as CD3+CD4−CD161hiVα7.2+ cells. However, some MAIT cells express CD4. It would be interesting to look at CD4 (and CD8) coreceptor expression if this data is available. 

Response: Thank you for pointing this out. We have examined the flow cytometry data with and without CD4 involved in the gating strategy and this did not change any of our findings (no differences between day 2 and day 30). To address comments above and also posed by other reviewers, to be more consistent with MAIT identification by immunohistochemistry, we have changed the definition of MAIT cells to not include CD4 (as noted in statement above). Unfortunately, at the time of this study, we did not stain cells with CD8 antibody. 

Remaining issues: OK. But data in figures does not appear to be changed.

Previous comment. 9. For Fig. 4, FACS plots showing β7 expression on peripheral MAIT and non-MAIT cells should be included. Β7 data from healthy control is also required. 

Response: Given the lack of integrin β7 data from controls, and the limited information gained, we have decided to remove these data from our manuscript. 

Remaining issues (minor): Integrin B7 Ab still appears in methods (line 123).

Previous comment. 21. The data in Fig 5E doesn’t make sense. Frequencies should add up to 100 (line 254- 259 in text).

Response: We apologize for this confusing figure. The Y axis is the absolute number of times (not percentage) of CDR3b with a particular amino acid length. This has been corrected in results (line 263 is updated to number instead of frequency), the y-axis title, and the figure legend (line 429) has been modified to, “x-axis shows length distribution of amino acids and y axis shows number of times CDR3β sequence was found with that amino acid length.” 

Remaining issues: The figure now makes sense. However, if the authors wish to compare LPL to PBMC CDR3b length (line 263-) they need to consider the percentage of sequences rather than the numbers with each length. It seems the numbers in LPL at d2 are lower than PBMC due to the total number of sequences rather than a different pattern of CDR3b length. In fact, according to the results in Fig 5E (new 4E), a similar pattern of CDR3β sequence seems to be observed in MAIT cells from LPL and PBMC, with most frequent 14 nucleotide length.

Reviewer #3: The revised manuscript is much improved, and many inconsistencies have been addressed or clarified. 

However, the resolution of the imunoflourescence pictures is still very poor, and they can’t be used to verify the conclusions made. The authors argue that the experiments were performed in 2013, and that the quality can’t be improved. If so, I would suggest that another set of biopsies are collected and evaluated using equipment that will yield pictures of sufficient quality to confirm the conclusions made previously.

Likewise, it is argued that as the experiments were performed before the MR1 tetramers became available, the authors can’t confirm the MR1 reactivity of the cells identified as MAIT cells. I would suggest a round of new FACS analyses using biopsies from cholera-infected or convalescent patients to confirm that the CD161+Va7.2+ cells detected in the duodenal mucosa are actually MR1-reactive.

--------------------

PLOS authors have the option to publish the peer review history of their article (what does this mean?). If published, this will include your full peer review and any attached files.

If you choose “no”, your identity will remain anonymous but your review may still be made public.

**Do you want your identity to be public for this peer review?** For information about this choice, including consent withdrawal, please see our Privacy Policy.

Reviewer #1: No

Reviewer #2: No

Reviewer #3: No

Figure Files:

While revising your submission, please upload your figure files to the Preflight Analysis and Conversion Engine (PACE) digital diagnostic tool, https://pacev2.apexcovantage.com. PACE helps ensure that figures meet PLOS requirements. To use PACE, you must first register as a user. Then, login and navigate to the UPLOAD tab, where you will find detailed instructions on how to use the tool. If you encounter any issues or have any questions when using PACE, please email us at figures@plos.org.

Data Requirements:

Please note that, as a condition of publication, PLOS' data policy requires that you make available all data used to draw the conclusions outlined in your manuscript. Data must be deposited in an appropriate repository, included within the body of the manuscript, or uploaded as supporting information. This includes all numerical values that were used to generate graphs, histograms etc.. For an example see here: http://www.plosbiology.org/article/info%3Adoi%2F10.1371%2Fjournal.pbio.1001908#s5.

Reproducibility:

To enhance the reproducibility of your results, we recommend that you deposit your laboratory protocols in protocols.io, where a protocol can be assigned its own identifier (DOI) such that it can be cited independently in the future. Additionally, PLOS ONE offers an option to publish peer-reviewed clinical study protocols. Read more information on sharing protocols at https://plos.org/protocols?utm_medium=editorial-email&utm_source=authorletters&utm_campaign=protocols

# Author comment: Author response to Decision Letter 1

**DOI:** 10.1371/journal.pntd.0010411.r004

17 Mar 2022

Attachment Submitted filename: pNTD 21-00600R1 Response to reviewers.pdf Click here for additional data file.

# Peer review: Decision Letter 2

**DOI:** 10.1371/journal.pntd.0010411.r005

**Authors:** Alfredo G Torres, Khitam Muhsen

11 Apr 2022

Dear Dr. Leung,

We are pleased to inform you that your manuscript 'Mucosal-Associated Invariant T (MAIT) Cells are Highly Activated in Duodenal Tissue of Humans withVibrio cholerae O1 Infection: A Preliminary Report' has been provisionally accepted for publication in PLOS Neglected Tropical Diseases.

Before your manuscript can be formally accepted you will need to complete some formatting changes, which you will receive in a follow up email. A member of our team will be in touch with a set of requests.

Please note that your manuscript will not be scheduled for publication until you have made the required changes, so a swift response is appreciated.

IMPORTANT: The editorial review process is now complete. PLOS will only permit corrections to spelling, formatting or significant scientific errors from this point onwards. Requests for major changes, or any which affect the scientific understanding of your work, will cause delays to the publication date of your manuscript.

Should you, your institution's press office or the journal office choose to press release your paper, you will automatically be opted out of early publication. We ask that you notify us now if you or your institution is planning to press release the article. All press must be co-ordinated with PLOS.

Thank you again for supporting Open Access publishing; we are looking forward to publishing your work in PLOS Neglected Tropical Diseases.

Best regards,

Khitam Muhsen

Guest Editor

PLOS Neglected Tropical Diseases

Alfredo Torres

Deputy Editor

PLOS Neglected Tropical Diseases

***********************************************************

The authors addressed well all comments raised by the reviewers and editors.

Reviewer's Responses to Questions

**Key Review Criteria Required for Acceptance?**

As you describe the new analyses required for acceptance, please consider the following:

**Methods**

-Are the objectives of the study clearly articulated with a clear testable hypothesis stated?

-Is the study design appropriate to address the stated objectives?

-Is the population clearly described and appropriate for the hypothesis being tested?

-Is the sample size sufficient to ensure adequate power to address the hypothesis being tested?

-Were correct statistical analysis used to support conclusions?

-Are there concerns about ethical or regulatory requirements being met?

Reviewer #1: The relatively small sample size is an overall weakness, but it relates to the difficulties in obtaining human tissues (e.g., ethical, and logistical issues). The small sample size might have precluded the authors from rejecting the null hypothesis, and this shortcoming is now clearly explained in the discussion section of the revised version. The readers will now be able to judge the weaknesses and shortcomings of the present work, and the number of citations received by this manuscript, if published, will demonstrate its importance.

Reviewer #2: (No Response)

**********

**Results**

-Does the analysis presented match the analysis plan?

-Are the results clearly and completely presented?

-Are the figures (Tables, Images) of sufficient quality for clarity?

Reviewer #1: It has been a “tour de force” catching up on the many typos and inconsistencies in this manuscript. It is also a wonder how many of these issues could escape the attention of a large group of co-authors. Nevertheless, the manuscript is sound, interesting, and presents novel findings, fulfilling the PLOS NTD mission of publishing findings that improve the understanding of diseases affecting developing countries using a relevant human tissue model.

Reviewer #2: (No Response)

**********

**Conclusions**

-Are the conclusions supported by the data presented?

-Are the limitations of analysis clearly described?

-Do the authors discuss how these data can be helpful to advance our understanding of the topic under study?

-Is public health relevance addressed?

Reviewer #1: Although all the points raised by this reviewer have been addressed satisfactorily since the first revision, all the new changes/additions clearly add to the contribution of this paper.

Reviewer #2: (No Response)

**********

**Editorial and Data Presentation Modifications?**

Use this section for editorial suggestions as well as relatively minor modifications of existing data that would enhance clarity. If the only modifications needed are minor and/or editorial, you may wish to recommend “Minor Revision” or “Accept”.

Reviewer #1: I recommend an acceptance, though it would be great to have Figure 1A removed or cropped to better visualize the findings.

Reviewer #2: (No Response)

**********

**Summary and General Comments**

Use this section to provide overall comments, discuss strengths/weaknesses of the study, novelty, significance, general execution and scholarship. You may also include additional comments for the author, including concerns about dual publication, research ethics, or publication ethics. If requesting major revision, please articulate the new experiments that are needed.

Reviewer #1: Although all the points raised by this reviewer have been addressed satisfactorily since the first revision, all the new changes/additions clearly add to the contribution of this paper.

Reviewer #2: No further comments.

**********

PLOS authors have the option to publish the peer review history of their article (what does this mean?). If published, this will include your full peer review and any attached files.

If you choose “no”, your identity will remain anonymous but your review may still be made public.

**Do you want your identity to be public for this peer review?** For information about this choice, including consent withdrawal, please see our Privacy Policy.

Reviewer #1: No

Reviewer #2: No

# Editor report: Acceptance letter

**DOI:** 10.1371/journal.pntd.0010411.r006

**Authors:** Alfredo G Torres, Khitam Muhsen

30 Apr 2022

Dear Dr. Leung,

We are delighted to inform you that your manuscript, "Mucosal-Associated Invariant T (MAIT) Cells are Highly Activated in Duodenal Tissue of Humans withVibrio cholerae O1 Infection: A Preliminary Report," has been formally accepted for publication in PLOS Neglected Tropical Diseases.

We have now passed your article onto the PLOS Production Department who will complete the rest of the publication process. All authors will receive a confirmation email upon publication.

The corresponding author will soon be receiving a typeset proof for review, to ensure errors have not been introduced during production. Please review the PDF proof of your manuscript carefully, as this is the last chance to correct any scientific or type-setting errors. Please note that major changes, or those which affect the scientific understanding of the work, will likely cause delays to the publication date of your manuscript. Note: Proofs for Front Matter articles (Editorial, Viewpoint, Symposium, Review, etc...) are generated on a different schedule and may not be made available as quickly.

Soon after your final files are uploaded, the early version of your manuscript will be published online unless you opted out of this process. The date of the early version will be your article's publication date. The final article will be published to the same URL, and all versions of the paper will be accessible to readers.

Thank you again for supporting open-access publishing; we are looking forward to publishing your work in PLOS Neglected Tropical Diseases. 

Best regards,

Shaden Kamhawi

co-Editor-in-Chief

PLOS Neglected Tropical Diseases

Paul Brindley

co-Editor-in-Chief

PLOS Neglected Tropical Diseases
